# Endoplasmic reticulum–resident **α**-glucosidase II drives non-small cell lung cancer progression via regulation of secretory glycoproteins

**DOI:** 10.1172/jci.insight.203262

**Published:** 2026-06-08

**Authors:** Shike Wang, Na Ding, Angelo Chen, Derrick Cardin, Yuting Xu, Kate Grimley, William K. Russell, Jun Xu, Jonathan M. Kurie, Guan-Yu Xiao, Xiaochao Tan

**Affiliations:** 1 Department of Integrative Biology and Pharmacology, The University of Texas Health Science Center at Houston, Texas, USA.; 2 Department of Thoracic/Head and Neck Medical Oncology, The University of Texas MD Anderson Cancer Center, Houston, Texas, USA.; 3 Department of Biosciences, Rice University, Houston, Texas, USA.; 4 Department of Medicine, Section of Hematology Oncology, Tulane University School of Medicine, Louisiana Cancer Research Center, New Orleans, Louisiana, USA.; 5 Hillsdale College, Hillsdale, Michigan, USA.; 6 Department of Biochemistry & Molecular Biology, The University of Texas Medical Branch at Galveston, Houston, Texas, USA.; 7 Molecular and Cellular Biology Department, The Advanced Cell Engineering and 3D Models Core, Baylor College of Medicine, Houston, Texas, USA.; 8 Department of Toxicology and Cancer Biology, The University of Kentucky, Lexington, Kentucky, USA.

**Keywords:** Cell biology, Oncology, Cancer, Lung cancer, Protein traffic

## Abstract

Non-small cell lung cancer (NSCLC) remains a leading cause of cancer-related mortality worldwide, yet its molecular drivers are not fully defined. Emerging evidence highlights the importance of tumor-stroma interactions mediated by secreted glycoproteins. However, the mechanisms by which cancer cells regulate the secretion of these protumorigenic proteins remain largely unknown. Endoplasmic reticulum–resident (ER-resident) N-glycan–processing enzymes regulate proper protein folding, a prerequisite for glycoproteins to exit the ER and undergo secretion. By evaluating their prognostic significance in lung tumors and conducting functional screening in lung cancer cells, we identify α-glucosidase II (α-Glc II) as a key regulator of NSCLC progression. α-Glc II promotes tumor growth and dissemination in a glucosidase activity–dependent manner in orthotopic mouse lung tumor model. Genetic disruption of α-Glc II induced ER stress and reduced cell proliferation and motility. Mechanistically, α-Glc II–mediated N-glycan modification regulated the ER-to-Golgi trafficking and secretion of specific oncogenic glycoproteins, including lysyl hydroxylase 2 (LH2), Tissue Inhibitor of Metalloproteinase 1 (TIMP1), and TGF-β, which are known to be associated with extracellular matrix remodeling. These findings uncover a role for ER glycosylation machinery in shaping the NSCLC secretome and highlight α-Glc II as a potential therapeutic target.

## Abstract 2

α-glucosidase II promotes the malignancy of Lung Adenocarcinoma by regulating N-glycan processing and the secretion of its substrates, suggesting that it may serve as a potential therapeutic target for lung cancer.

## Graphical abstract

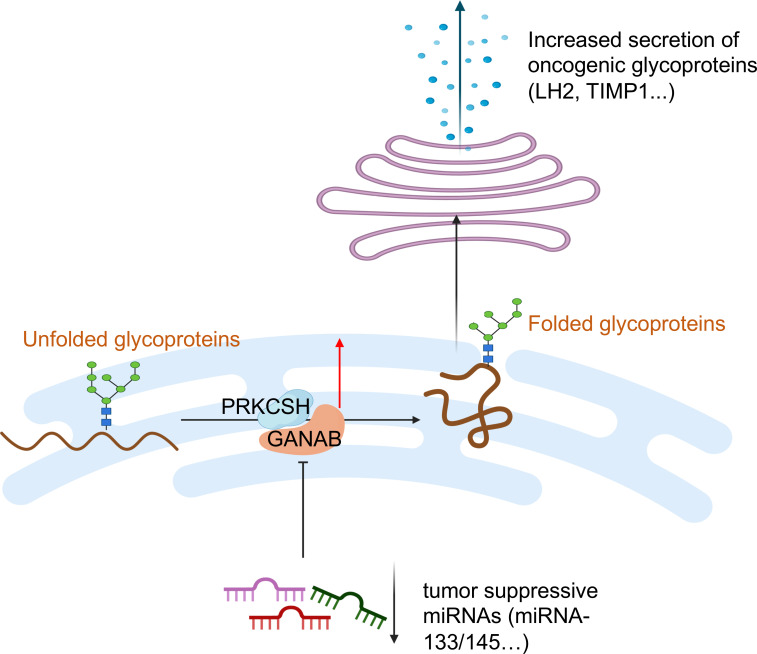

## Introduction

Non-small cell lung cancer (NSCLC), which includes lung adenocarcinoma (LUAD), lung squamous cell carcinoma (LUSC), and large cell lung carcinoma, accounts for ~85% of lung cancers and remains a leading cause of cancer-related death worldwide despite advances in targeted and immune therapies (1, 2). While significant progress has been made in understanding the genetic drivers of NSCLC, such as mutations in *EGFR*, *KRAS*, and *ALK*, increasing evidence suggests that nongenetic mechanisms — particularly those involving the tumor microenvironment (TME) — play a critical role in promoting tumor growth and metastasis (3–5).

A central feature of tumor progression is the dynamic remodeling of the TME through the secretion of cytokines, growth factors, extracellular matrix (ECM) components, and enzymes from cancer cells that regulate tumor cell adhesion, migration, and immune evasion (6–8). The secretion and function of these proteins are tightly regulated by posttranslational modifications, including N-linked glycosylation, which is essential for protein folding, trafficking, and stability (9). N-glycan modification is a key step in the quality control of glycoproteins within the endoplasmic reticulum (ER). After the transfer of a precursor oligosaccharide to nascent polypeptides, the glycan is sequentially trimmed by ER-resident enzymes, including α-glucosidase I (α-Glc I) and α-glucosidase II (α-Glc II) (10, 11). This processing generates monoglucosylated N-glycans that serve as recognition signals for the lectin chaperones calnexin and calreticulin, which promote proper protein folding and subsequent ER exit (10, 11). This tightly regulated machinery selectively directs appropriately folded proteins through the secretory pathway, thereby maintaining ER proteostasis and preventing the secretion of misfolded proteins. Disruption of ER glycosylation pathways has been implicated in cancers (12), but the roles of ER-resident enzymes in shaping the cancer cell–derived secretome during NSCLC progression remain incompletely understood.

In this study, we found several ER-resident enzymes involving in N-glycan processing are frequently upregulated in human cancers. Among them, α-Glc II is a highly conserved enzyme that catalyzes the sequential removal of glucose residues from N-linked glycans — a critical step in glycoprotein maturation and ER quality control. α-Glu II is composed of a catalytic α-subunit encoded by GANAB and a noncatalytic β-subunit encoded by PRKCSH; the latter facilitates substrate binding and the α-subunit stabilization (13). PRKCSH deficiency inhibits growth and metastatic potential of lung cancer cells by reducing receptor tyrosine kinase activities (14) and promotes an antitumor immune microenvironment via unfolded protein response activation (15). However, the role of GANAB and the mechanisms underlying the tumor-promoting function of α-Glc II in lung cancer remain unclear.

By analyzing expression levels and prognostic significance in lung tumors, and using in vitro proliferation, migration, and invasion assays alongside in mouse tumor growth models, we found that GANAB plays a critical role in lung cancer cell growth and motility. Unbiased proteomic analysis further identified the secretome regulated by GANAB and α-Glc II. These findings reveal a previously unrecognized mechanism by which cancer cells remodel N-glycosylation to control the secretion of oncogenic glycoproteins.

## Results

### N-glycan modification is crucial to lung cancer progression.

**Figure 1:**
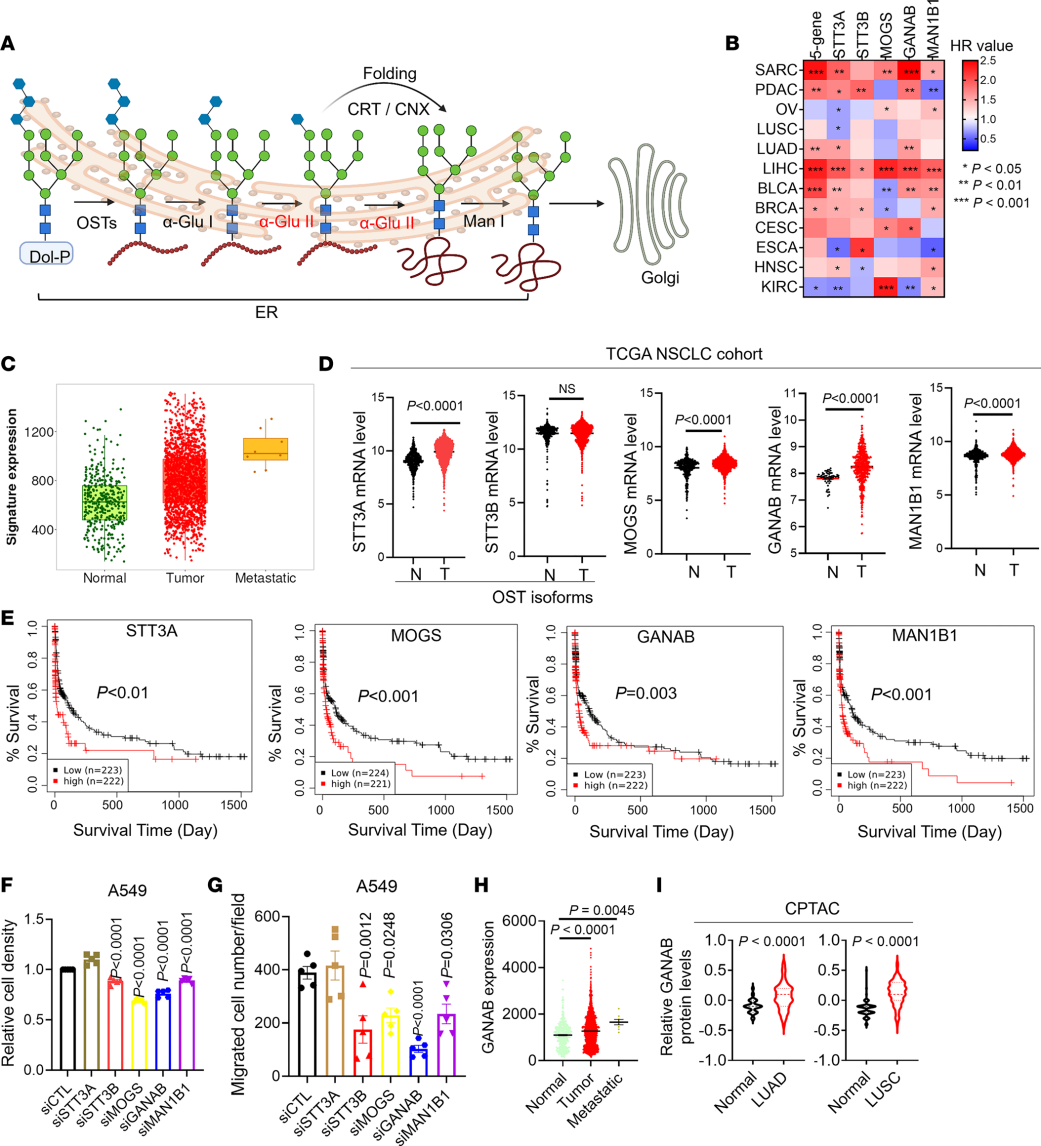
N-glycan–modifying enzymes are upregulated in NSCLC. (**A**) Overview of N-linked glycosylation in the endoplasmic reticulum (ER). The Glc3Man9GlcNAc2 glycan is transferred from its lipid-linked donor, dolichol phosphate–Glc3Man9GlcNAc2, to an asparagine (Asn) residue within the consensus sequence Asn-X-Ser/Thr of a nascent protein by the oligosaccharyltransferase (OST) complex. The glycan is then sequentially trimmed by α-glucosidase I (α-Glc I), α-glucosidase II (α-Glc II), and mannosidase I (Man I) in the ER. Once properly folded, the glycoproteins are transported to the Golgi. (**B**) Analysis of mRNA expression levels of genes encoding N-glycan–modifying enzymes across multiple cancer types in the TCGA cohort. *P* values were analyzed using cox proportional hazards model. SARC, sarcoma; PDAC, pancreatic ductal adenocarcinoma; OV, ovarian serous cystadenocarcinoma; LUSC, lung squamous cell carcinoma; LUAD, lung adenocarcinoma; LIHC, liver hepatocellular carcinoma; BLCA, bladder urothelial carcinoma; BRCA, breast invasive carcinoma; CESC, cervical and endocervical cancers; ESCA, esophageal carcinoma; HNSC, head and neck squamous cell carcinoma; KIRC, kidney renal clear cell carcinoma. (**C**) Expression levels of a 5-gene signature (STT3A, STT3B, MOGS, GANAB, and MAN1B1) in normal lung tissues (*n* = 391), primary lung tumors (*n* = 1865), and distant metastases (*n* = 8) using data from TNMplot (https://tnmplot.com). (**D**) mRNA levels of genes encoding N-glycan–modifying enzymes in the TCGA NSCLC cohort. (**E**) Kaplan-Meier survival analysis of patients with LUAD based on individual gene expression level above (high) or below (low) the median value using GENT2 (http://gent2.appex.kr/gent2/) database. *P* values were calculated using the log-rank test. (**F**) Relative cell densities by WST-1 proliferation assays. (**G**) Boyden chamber assays of migratory activity. (**H**) mRNA levels of GANAB in normal lung tissues (*n* = 391), primary lung tumors (*n* = 1865), and distant metastases (*n* = 8) using data from TNMplot (https://tnmplot.com/). (**I**) Relative GANAB protein levels in the Clinical Proteomic Tumor Analysis Consortium (CPTAC) database. *P* values were determined using 2-tailed Student’s *t* test (**D** and **I**) or 1-way ANOVA (**F**–**H**). Data indicate the mean ± SEM from a single experiment incorporating biological replicate samples (*n* ≥ 3) and are representative of at least 2 independent experiments.

ER-resident N-glycan modifying enzymes controls the folding, stability, and trafficking of glycoproteins (Figure 1A). To investigate whether these enzymes are involved in human cancers, we first analyzed the mRNA levels of these genes (STT3A/STT3B encoding the catalytic subunits of the oligosaccharyltransferase complex; MOGs encoding glucosidase I; GANAB encoding the catalytic subunit of glucosidase II; MAN1B1 encoding ER Mannosidase I) across multiple cancer types using The Cancer Genome Atlas (TCGA) data and found they were frequently upregulated across various cancers (Figure 1B). In lung cancer specifically, the expression signature of these 5 genes was elevated in both primary tumor tissues and metastatic sites compared with normal tissues (Figure 1C). Notably, all genes — except STT3B — were upregulated in the TCGA NSCLC cohort (Figure 1D), and their higher expression levels were associated with shorter overall survival in lung cancer patients (Figure 1E), supporting a potential role for the N-glycan modifying enzymes in NSCLC.

To functionally evaluate this, we utilized small interfering RNAs (siRNAs) to knock down each of these genes in A549 NSCLC cells (Supplemental Figure 1A; supplemental material available online with this article; https://doi.org/10.1172/jci.insight.203262DS1). Most gene knockdowns significantly reduced cell proliferation and migration (Figure 1, F and G, and Supplemental Figure 1B). Among them, the depletion of GANAB showed the most profound effect, which is consistent with that α-Glc II acts as a key factor in the N-glycan modification process (16, 17). GANAB mRNA expression was positively correlated with that of other N-glycan–related genes (Supplemental Figure 1C), suggesting they were coexpressed in NSCLC. Given GANAB’s central role in the quality control of secretory proteins, we prioritized it for the subsequent studies. GANAB expression was elevated in primary lung tumors compared with normal lung tissue and further increased in metastatic lesions (Figure 1H). Similarly, proteomic data from the CPTAC database further confirmed elevated GANAB protein levels in LUAD and LUSC, compared with normal tissues (Figure 1I). In addition, GANAB protein levels were higher in NSCLC cell lines compared with noncancerous epithelial cells (Supplemental Figure 2A). Collectively, these findings highlight the importance of N-glycan–modifying enzymes in NSCLC progression and identified GANAB as a potential regulator of NSCLC progression.

### GANAB expression is regulated by tumor suppressive microRNAs (miRNAs).

**Figure 2:**
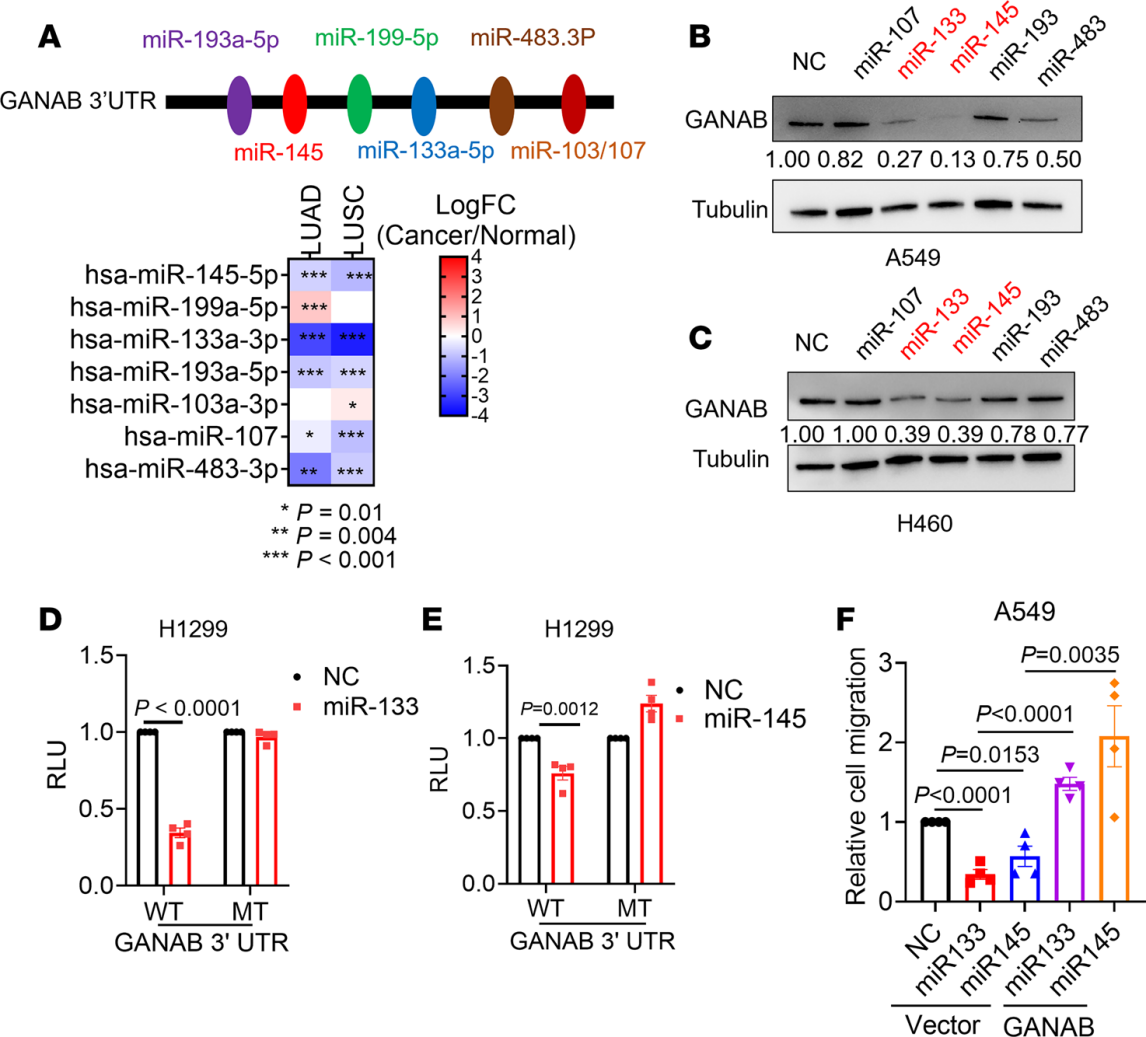
GANAB is a direct target of tumor-suppressive microRNAs (miRNAs). (**A**) Graph showing predicted miRNAs binding sites in the 3′ UTR of GANAB (top) and a heatmap showing the relative expression levels of these miRNAs in lung cancer tissues compared with normal tissues (bottom). (**B** and **C**) WB analysis of GANAB protein levels in A549 (**B**) and H460 (**C**) cells following ectopic expression of indicated miRNAs. Densitometric analysis was performed using ImageJ. (**D** and **E**) GANAB 3′-UTR reporter assays. H1299 cells were cotransfected with miR-133a mimics (**D**) or miR-145 mimics (**E**) and luciferase reporters containing either WT or miR-133a/miR-145 binding site–mutant 3′-UTRs of GANAB. *P* values were determined using 2-tailed Student’s *t* test. (**F**) Boyden chamber transwell assay on A549 cells cotransfected of miR-133a, miR-145, or negative control (NC) mimics and GANAB expression vector or control vector. *P* values were determined using 1-way ANOVA. Data indicate the mean ± SEM from a single experiment incorporating biological replicate samples (*n* ≥ 3) and are representative of at least 2 independent experiments.

To explore the underlying mechanism of GANAB upregulation in cancer, we first evaluated its transcriptional activity using a RNA Polymerase II chromatin immunoprecipitation assay. No significant difference in RNA Polymerase II occupancy at the GANAB promoter region was observed between noncancerous and NSCLC cell lines (Supplemental Figure 2B), suggesting that GANAB upregulation in NSCLC is unlikely due to enhanced transcription. miRNAs are a class of small noncoding RNAs that suppress the expression of their targets posttranscriptionally (18), and loss of tumor-suppressive miRNAs promotes the expression of oncogenic target genes (19). By analyzing the 3′ untranslated region (3′UTR) of GANAB, we found predicted bindings sites for several miRNAs (Figure 2A). Except for miR-199-5a and miR-103a-3p, all predicted miRNAs showed lower expression in NSCLC than in normal lung tissues (Figure 2B). Western blot (WB) analysis confirmed that ectopic expression of miR-133a and miR-145 remarkably reduced GANAB protein levels (Figure 2, C and D), suggesting that GANAB is regulated by these miRNAs. Treatment with miR-133a or miR-145 mimics suppressed the activity of GANAB reporters containing WT but not mutant 3′-UTRs lacking predicted miRNA binding sites, confirming that GANAB is a direct target of them (Figure 2, E and F). Furthermore, the inhibitory effects of miR-133a or miR-145 on cell migration were abolished by ectopic GANAB expression (Figure 2F). Together, these results demonstrate that GANAB upregulation in NSCLC is driven by reduced miRNA-mediated repression.

### α-Glc II activity is essential for NSCLC progression.

**Figure 3:**
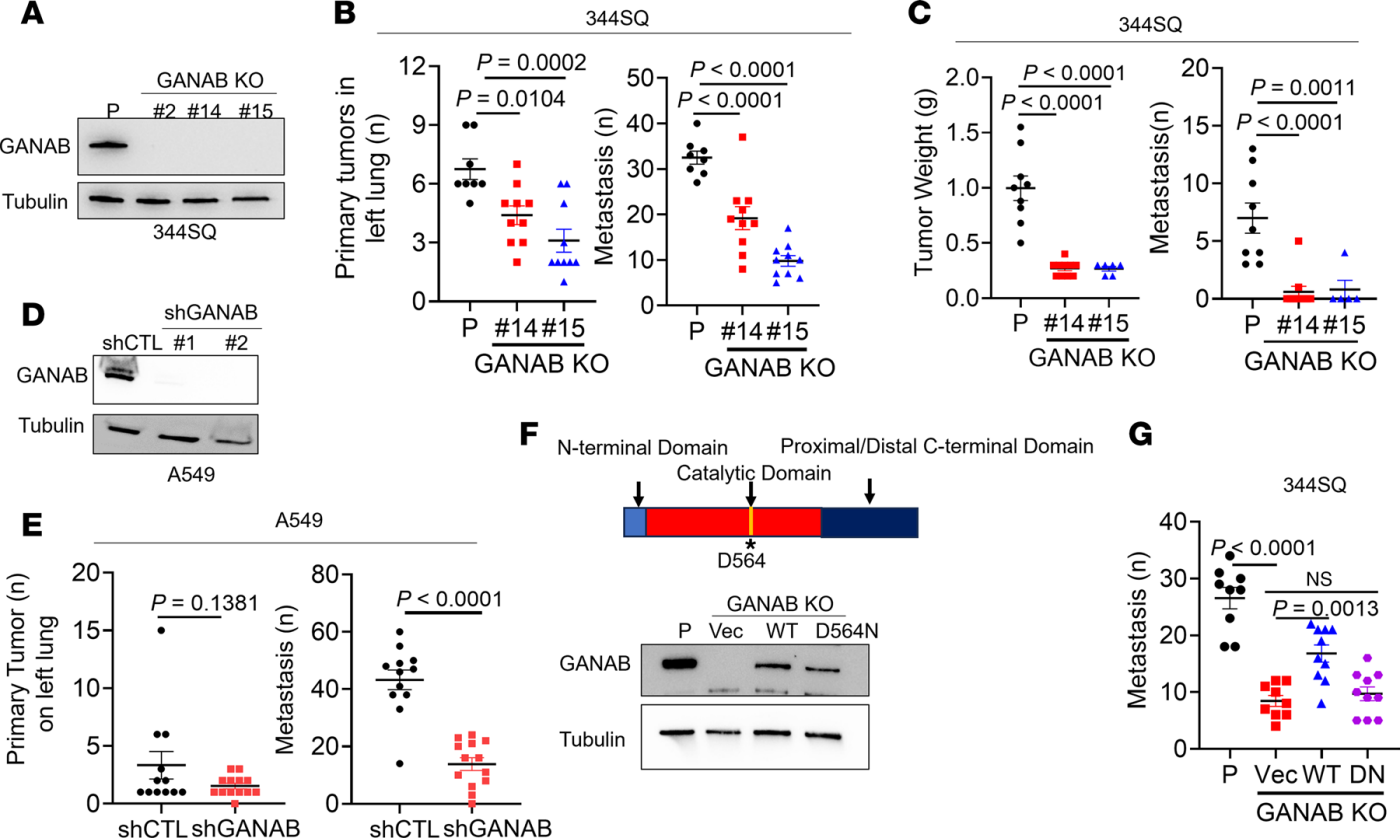
GANAB promotes tumor growth and metastasis in a enzymatic activity-dependent manner. (**A**) Validation of CRISPR-CAS9 mediated GANAB knockout (KO) in 344SQ cells by WB. P, parental cells. (**B**) Numbers of orthotopic lung tumors (left) and metastases to mediastinal nodes and contralateral lung (right) in syngeneic, immunocompetent mice (129/SV) (dots represent individual mouse numbers) injected orthotopically with P or GANAB-KO 344SQ cells. *P* values were determined using 1-way ANOVA. (**C**) Flank tumor weights (left) and numbers of metastases to mediastinal nodes and contralateral lung (right) in 129/SV mice (dots represent individual mouse numbers) injected s.c. with P or GANAB-KO 344SQ cells. *P* values were determined using 1-way ANOVA. (**D**) WB confirmation of GANAB expression in A549 cells stably transfected with shRNAs targeting GANAB (shGANAB) or control shRNA (shCTL). (**E**) Numbers of orthotopic lung tumors (left) and metastases to mediastinal and contralateral lung (right) in nude mice (dots represent individual mouse numbers) injected orthotopically with shCTL- or shGANAB-A549 cells. *P* values were determined using Student’s *t* test. (**F**) Top: Schematic diagram of GANAB domains highlighting the catalytic inactive site (D564). Bottom: WB confirmation of GANAB expression in P- and GANAB-KO–344SQ cells reconstituted without (Vec) or with WT GANAB or D564N mutant GANAB. (**G**) Numbers of orthotopic lung tumor metastases to mediastinal nodes and contralateral lung in 129/SV mice (dots represent individual mouse numbers) injected orthotopically with P- or GANAB-KO–344SQ cells reconstituted with WT or mutant (D564N) GANAB. *P* values were determined using 1-way ANOVA. Data indicate the mean ± SEM from a single experiment incorporating biological replicate samples (*n* ≥ 3) and are representative of at least 2 independent experiments.

Given that GANAB is the catalytic subunit of α-Glc II (13), a key regulator of N-glycan modification, we next sought to determine the role of GANAB-mediated α-Glc II activity in NSCLC progression. We disrupted this process by depleting GANAB using either CRISPR/Cas9-mediated KO or stable short-hairpin RNA–mediated (shRNA-mediated) knockdown in 344SQ cells (Figure 3A and Supplemental Figure 3A), a murine LUAD cell line derived from Kras^G12D^- and Trp53^R172H^-driven mouse LUAD tumors (20). In both orthotopic and s.c. models, GANAB depletion significantly reduced primary tumor growth and metastasis (Figure 3, B and C, and Supplemental Figure 3, B and C). A similar inhibitory effect was also found in human LUAD A549 cells following GANAB depletion (Figure 3, D and E). To rule out off-target effects, we reconstituted GANAB expression in GANAB-KO 344SQ cells (Figure 3F). Notably, only WT GANAB, but not the catalytically inactive mutant (D564N) (21), restored the metastatic capacity (Figure 3G), indicating that the α-Glc II activity is required for lung cancer progression.

**Figure 4:**
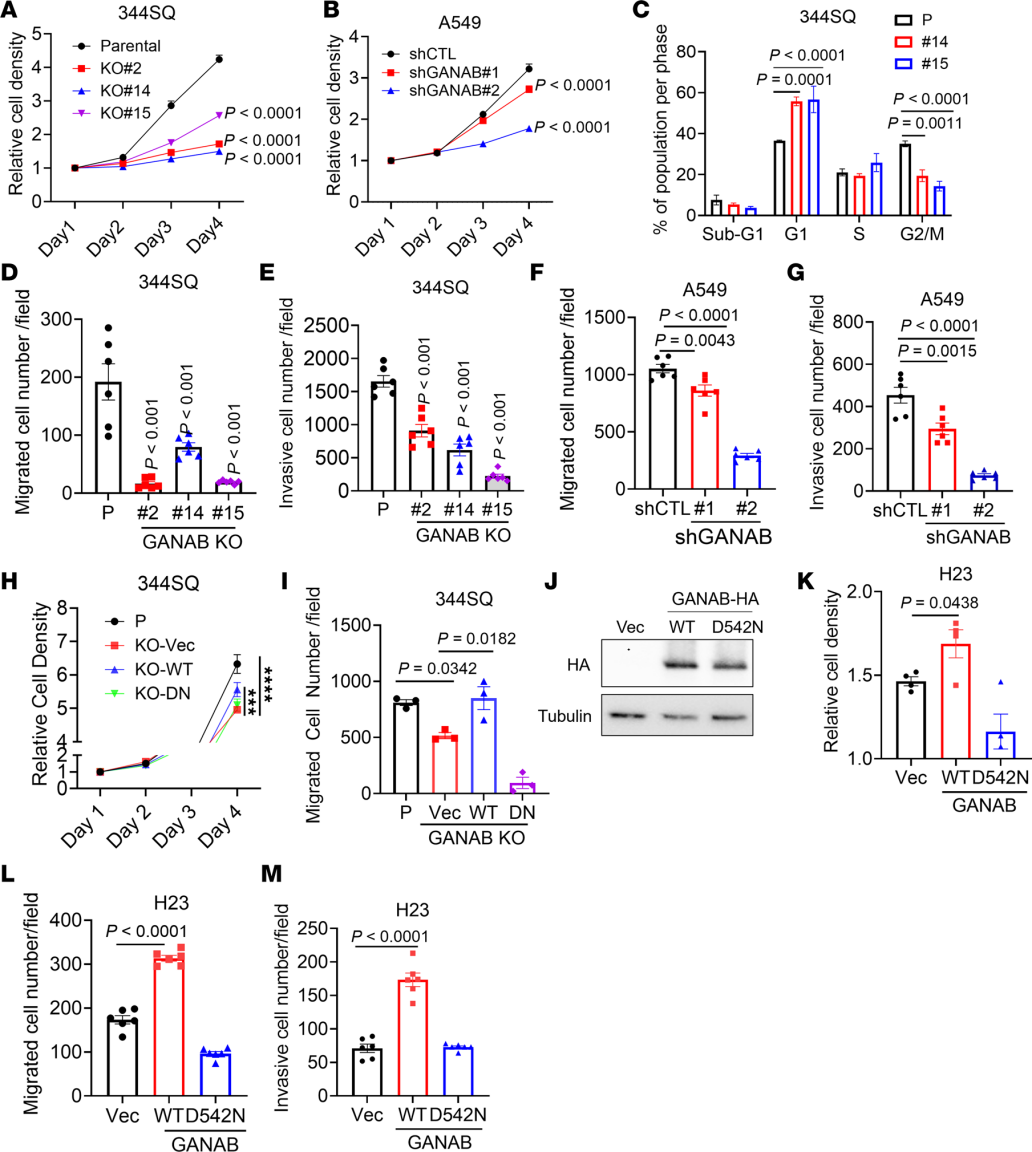
GANAB regulates lung cancer cell proliferation and motility. (**A** and **B**) Relative cell densities measured by WST-1 proliferation assays in 344SQ (**A**) and A549 (**B**) cells. (**C**) Cell cycle analysis was performed on P- and GANAB-KO–344SQ cells. *P* value was analyzed using 1-way ANOVA. (**D**–**G**) Boyden chamber transwell assays assessing migratory (**D** and **F**) and invasive (**E** and **G**) activity. (**H**) Relative cell densities measured by WST-1 proliferation assays. (**I**) Boyden chamber transwell assays assessing cell migratory activity. (**J**) WB confirmation of ectopic expression of WT or D542N mutant GANAB in H23 cells. (**K**) Relative cell densities measured by WST-1 proliferation assays. (**L** and **M**) Boyden chamber assays assessing migratory (**L**) and invasive (**M**) activity. *P* values were determined using 2-way ANOVA (**A**, **B**, and **H**) or 1-way ANOVA (**C**–**G**, **I**, and **K**–**M**). Data indicate the mean ± SEM from a single experiment incorporating biological replicate samples (*n* ≥ 3) and are representative of at least 2 independent experiments.

To determine the way in which α-Glu II regulates lung cancer progression, we first evaluated the effect of GANAB depletion on cell growth and found that GANAB depletion impedes cell proliferation significantly (Figure 4, A and B, and Supplemental Figure 4). Cancer cell growth is primarily driven by alterations in cell cycle regulation and evasion of apoptosis (22). Interestingly, GANAB depletion did not induce apoptosis in monolayer cultures (Supplemental Figure 5, A–D). Instead, it led to marked G1 phase cell cycle arrest (Figure 4C and Supplemental Figure 5E), suggesting that GANAB promotes cancer cell growth mainly through regulating cell cycle progression. Boyden chamber assays revealed that GANAB depletion significantly reduced the migratory and invasive capacities of NSCLC cells (Figure 4, D–G, and Supplemental Figure 6, A–H). Notably, disruption of GANAB in BEAS-2B cells did not significantly affect cell motility (Supplemental Figure 6, I–K), suggesting that noncancerous cells are less dependent on α-Glc II function. Moreover, consistent with the in vivo results, WT GANAB — but not the catalytically inactive mutant — was able to restore the proliferation and migration abilities of GANAB-depleted cells (Figure 4, H and I). Similarly, ectopic expression of WT GANAB in NSCLC cell lines with low endogenous GANAB expression (H23, H1299) was sufficient to promote both cell growth and migration, whereas the catalytic inactive mutant was unable to recapitulate these phenotypes (Figure 4, J–M, and Supplemental Figure 7, A–C), confirming the essential role of α-Glu II catalytic activity in promoting cancer cell growth and migration/invasion.

Knockdown of PRKCSH in human NSCLC cells (A549, H460) using either shRNA or siRNAs led to reduced α-subunit levels as expected, along with decreased cell proliferation, migration, and invasion (Supplemental Figure 8), closely mimicking the effects of GANAB depletion. These findings highlight the critical role of α-Glu II enzymatic activity in promoting NSCLC cancer progression.

### α-Glc II activity regulates ER homeostasis.

**Figure 5:**
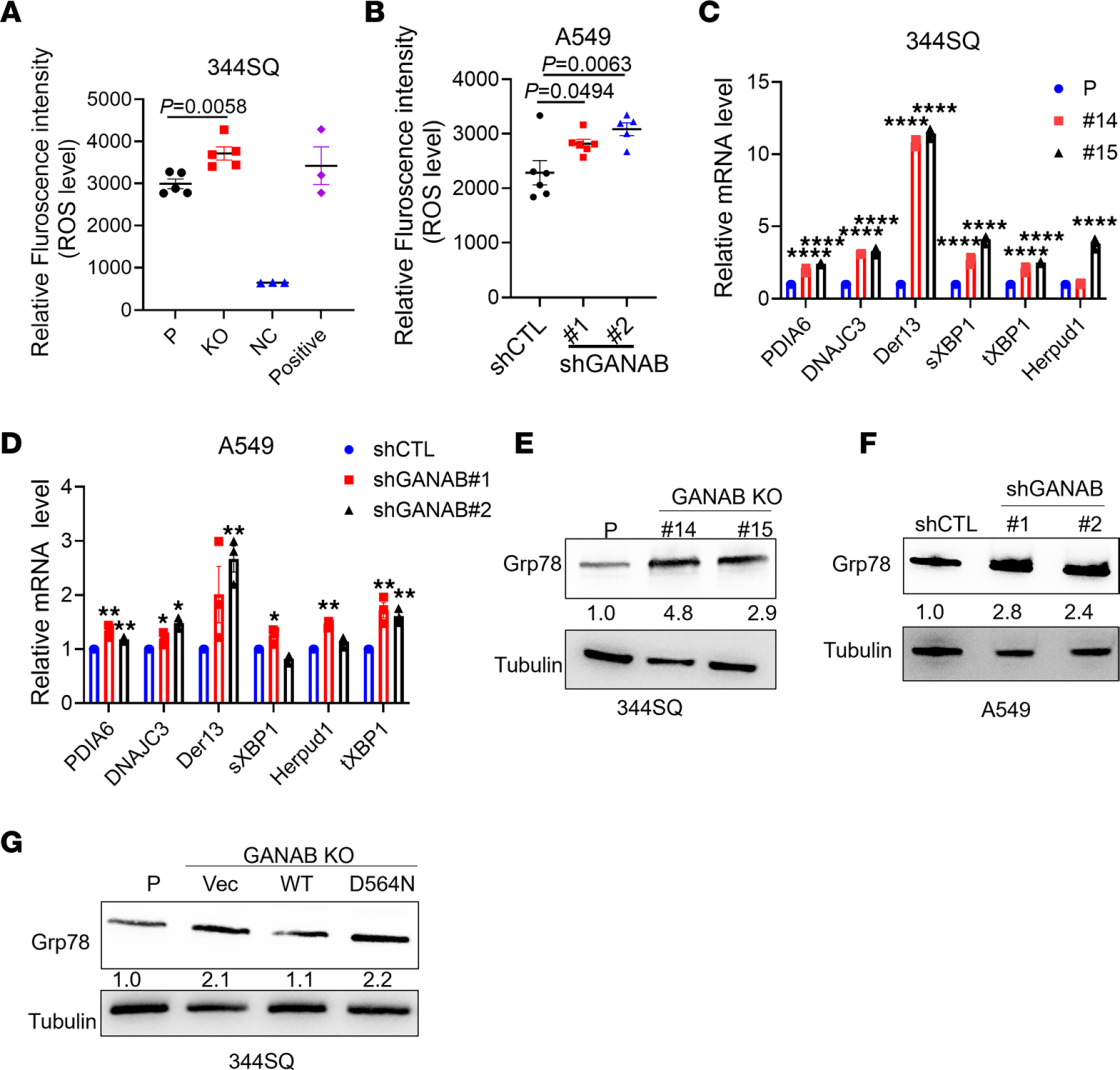
GANAB deficiency induces ER-stress. (**A**) ROS level analysis in P- and GANAB-KO–344SQ cells. Negative control (NC) and positive controls were included according to the manufacturer’s instructions for the ROS detection kit. (**B**) ROS level analysis in shCTL- and shGANAB-A549 cells. (**C**) qPCR analysis of target gene expression in P- and GANAB-KO–344SQ cells. (**D**) qPCR analysis of target gene expression in shCTL- and shGANAB-A549 cells. (**E**–**G**) WB confirmation of target protein levels in the indicated cell lines. *P* values were determined using 1-way ANOVA. *P<0.05; **P<0.01; ****P<0.0001. Data indicate the mean ± SEM from a single experiment incorporating biological replicate samples (*n* ≥ 3) and are representative of at least 2 independent experiments.

To identify the signaling pathways potentially regulated by α-Glu II, we performed RNA-seq analysis on parental and GANAB-depleted 344SQ cells. GANAB depletion led to the upregulation of over 1,000 genes and downregulation of approximately 700 genes (Supplemental Figure 9A). Gene Ontology (GO) analysis revealed that the downregulated genes were primarily associated with actin binding, growth factor binding, and ECM organization (Supplemental Figure 9B) — pathways closely linked to cell proliferation and motility, consistent with our earlier findings. Interestingly, GO analysis of the upregulated genes showed significant enrichment in oxidoreductase activity and glutathione transferase activity, both involved in cellular responses to oxidative stress (Supplemental Figure 9C) (23). We validated the upregulation of these genes in GANAB-depleted 344SQ and A549 cells by qPCR (Supplemental Figure 9, D and E). We thus hypothesized that disruption of α-Glu II activity may trigger oxidative stress. In support of this hypothesis, GANAB depletion significantly increased reactive oxygen species (ROS) production in both 344SQ and A549 cells (Figure 5, A and B). The elevated ROS levels may result from disrupted ER homeostasis. Supporting this notion, GO analysis indicated activation of the ER stress response pathway in GANAB-depleted cells, supporting the notion that GANAB deficiency leads to glycoprotein misfolding and accumulation in the ER, thereby triggering ER stress. qPCR analysis further confirmed the upregulation of ER stress–related genes following depletion of either GANAB or PRKCSH (Figure 5, C and D, and Supplemental Figure 9, F and G). Furthermore, we validated ER stress induction by measuring the protein levels of 78 KDa Glucose-Regulated Protein (GRP78), a well-established ER stress marker (24). Notably, the α-Glc II activity is essential for Grp78 reduction (Figure 5, E–G, and Supplemental Figure 9H), further supporting the role of α-Glu II in maintaining ER homeostasis.

### α-Glc II regulates a malignant secretome.

**Figure 6:**
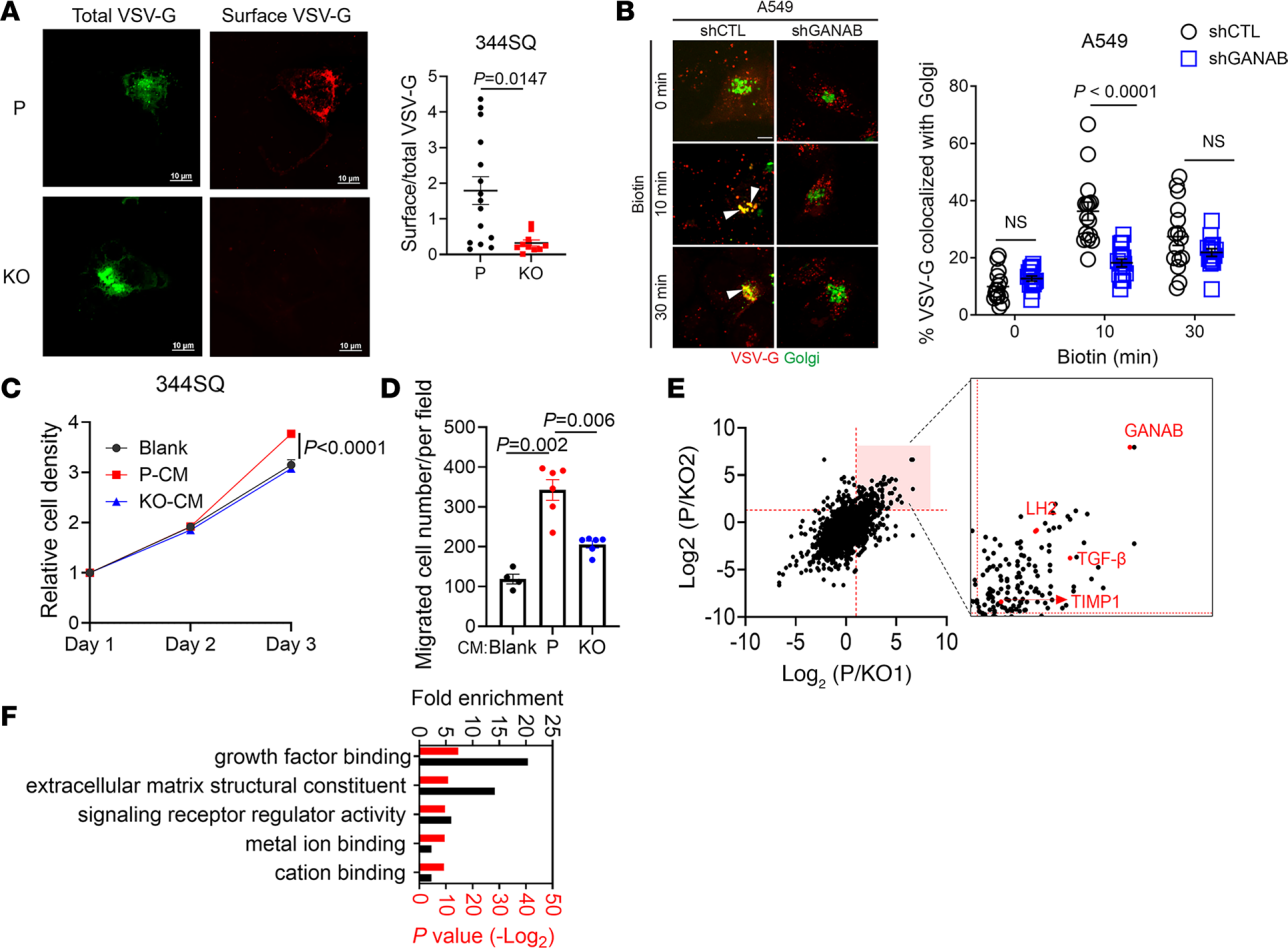
GANAB regulates secretion. (**A**) Representative images (left panel) and quantification (right panel) of VSV-G trafficking assay performed in P- and GANAB-KO–344SQ cells. *P* values were determined using 2-tailed Student *t* test. (**B**) Representative images (left panel) and quantification (right panel) of dynamic VSV-G trafficking assay performed in shCTL- and shGANAB-A549 cells. *P* values were determined using 2 tailed Student’s *t* test. (**C**) Relative cell densities measured by WST-1 proliferation assays in 344SQ cells cultured in either FBS-free medium or conditioned medium collected from parental or GANAB-KO–344SQ cells. *P* values were determined using 2-way ANOVA. (**D**) Boyden chamber assays assessing migratory activity in 344SQ cells cultured in either FBS-free medium or conditioned medium collected from parental or GANAB-KO–344SQ cells. *P* values were determined using 1-way ANOVA. (**E**) Dot plot showing reduced secreted proteins in CM of both GANAB-KO–344SQ clones compared with CM of parental 344SQ cells identified by LC-MS analysis. (**F**) GO analysis of overlapping downregulated proteins involved in molecular function, identified in the conditioned medium of both GANAB-KO–344SQ clones compared with parental cells. Data indicate the mean ± SEM from a single experiment incorporating biological replicate samples (*n* ≥ 3) and are representative of at least 2 independent experiments.

Considering the essential role of N-glycan modification in the correct folding and ER-to-Golgi trafficking of secreted glycoproteins, we examined the effects of α-Glu II–mediated N-glycan processing on secretory trafficking. GANAB depletion led to a marked reduction in vesicular stomatitis virus glycoprotein (VSV-G) transport to the plasma membrane, a widely used assay for evaluating secretory pathway activity (Figure 6A). Additionally, we observed a significantly reduced colocalization of VSV-G with the Golgi apparatus in GANAB-depleted cells using the Retention Using Selective Hooks (RUSH) system (Figure 6B), suggesting impaired ER-to-Golgi trafficking and reduced secretory flux. We then evaluated the functional effect of α-Glu II–mediated secretion on cancer cell behavior. Conditioned media (CM) collected from parental or GANAB-depleted 344SQ cells were applied to 344SQ cells. Notably, CM from GANAB-depleted cells showed a diminished ability to promote cell proliferation and migration compared with that from parental cells (Figure 6, C and D), indicating that α-Glu II influences the production of a functional secretome. To further characterize α-Glu II–driven secretome, we performed proteomic analysis on the CM from parental and GANAB-depleted 344SQ cells and found that GANAB depletion reduced the secretion of many proteins (Figure 6E, Supplemental Figure 10, and Supplemental Table 2). GO analysis of these proteins indicated functional enrichment in categories related to growth factor binding (e.g., TIMP1, TGF-β1, HTRA1) and ECM organization (e.g., LH2, LOX4, COL6A1), both of which are crucial for cancer cell proliferation and motility (Figure 6F).

### α-Glc II regulates the secretion of downstream substates.

**Figure 7:**
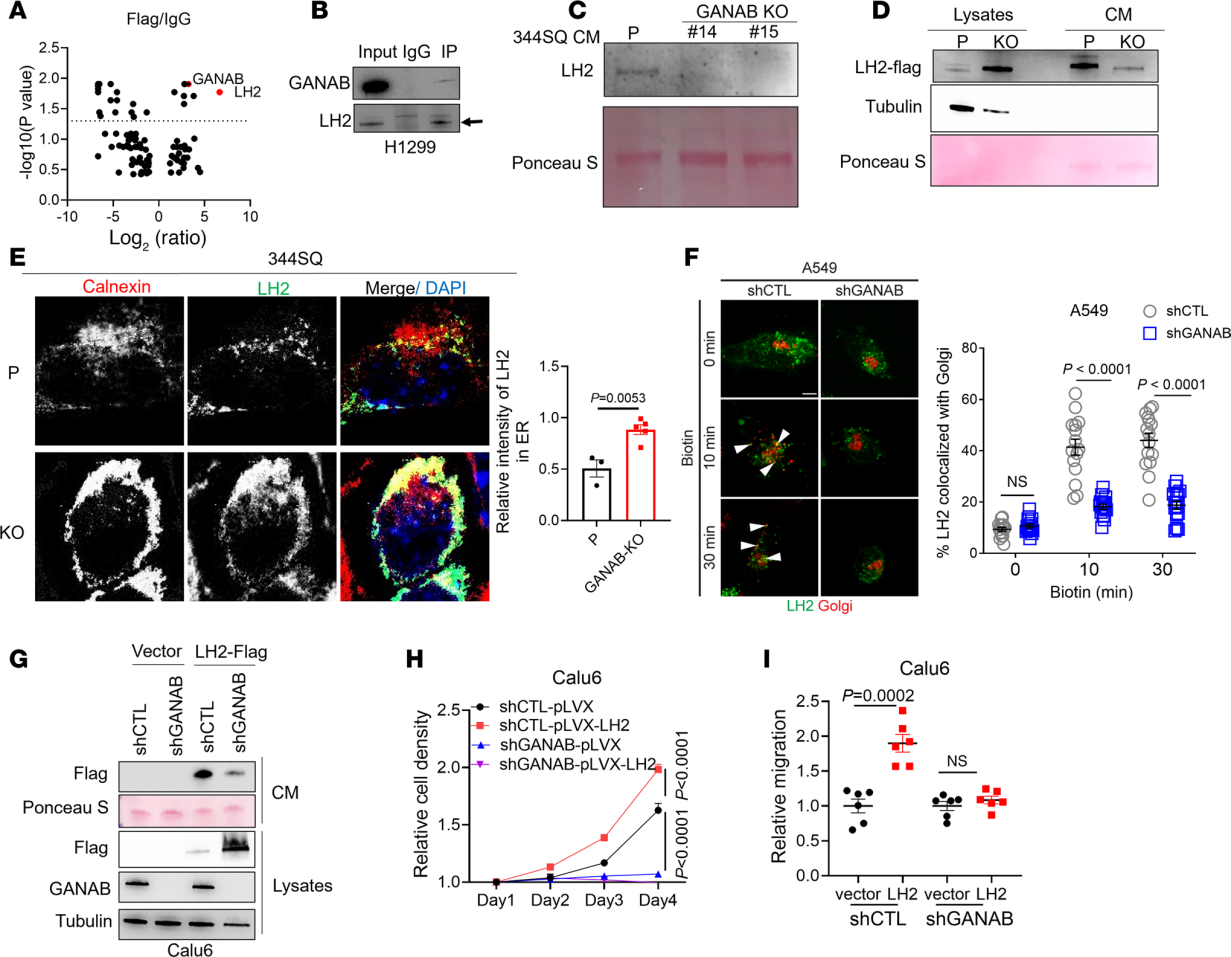
GANAB regulates the secretion and function of LH2. (**A**) Volcano plot of proteomic analysis by LC-MS of immunoprecipitation (IP) samples using LH2-Flag as bait, with IgG as a negative control. Differentially enriched proteins were identified based on a fold change ≥ 2 and adjusted *P* < 0.05. (**B**) WB analysis of target proteins in Co-IP samples using endogenous LH2 as bait in H1299 cells. (**C**) WB analysis of LH2 levels in conditioned medium from parental and GANAB-KO–344SQ cells. Ponceau S staining was used as a loading control. (**D**) WB analysis of exogenous LH2-Flag levels in cell lysates and conditioned medium from parental and GANAB-KO–344SQ cells. Tubulin served as the loading control for cell lysates, and Ponceau S staining was used as the loading control for conditioned medium. (**E**) Representative immunofluorescence images (top panel) showing LH2-Flag (green) and calnexin (red) staining, and relative intensity of LH2 within the ER normalized to total cellular LH2 intensity (right panel) in parental and GANAB KO 344SQ cells. Original magnification, 40×. *P* values were determined using 2-tailed Student *t* test. (**F**) Representative images (middle and bottom panel) and quantification (right panel) of the RUSH-LH2 dynamic trafficking assay in shCTL- and shGANAB-A549 cells. *P* values were determined using 2 tailed Student’s *t* test. (**G**) WB confirmation of ectopic LH2-Flag expression in shCTL- and shGANAB-Calu6 cell and secretion of LH2-Flag in CM. (**H**) Relative cell densities measured by WST-1 proliferation assays under the indicated conditions. *P* values were determined using 2-way ANOVA. (**I**) Boyden chamber assay assessing migratory activity under the indicated conditions. *P* values were determined using 2-tailed Student’s *t* test. Data indicate the mean ± SEM from a single experiment incorporating biological replicate samples (*n* ≥ 3) and are representative of at least 2 independent experiments.

We speculate that GANAB regulates secretion through interacting and modifying the glycoprotein substrates. Our previous unbiased proteomic analysis identified GANAB as a strong LH2 interactor (Figure 7A). Coimmunoprecipitation using endogenous LH2 as a bait confirmed the interaction between LH2 and GANAB (Figure 7B). Notably, secretion of both endogenous LH2 and ectopically expressed Flag-tagged LH2 was markedly reduced in GANAB-depleted cells (Figure 7, C and D). Meanwhile, LH2 accumulated within the cell lysates of GANAB-depleted cells (Figure 7D). Immunofluorescence staining of exogenous LH2 revealed its accumulation in the ER in GANAB-depleted 344SQ cells (Figure 7E). To directly monitor LH2 trafficking, we employed the RUSH system and found the ER-to-Golgi trafficking of LH2 was significantly impaired in GANAB-depleted A549 cells (Figure 7F). Importantly, ectopic expression of LH2 promoted cell proliferation and migration in GANAB-repleted but not -depleted CALU6 NSCLC cells (Figure 7, G–I), indicating that LH2 requires α-Glu II activity for its proper function. Similarly, TIMP1, another candidate identified in the GANAB-regulated secretome, was found to interact with GANAB (Supplemental Figure 11A). GANAB depletion reduced TIMP1 secretion and caused its accumulation in the ER (Supplemental Figure 11, B, and C). Like LH2, the proproliferative effect of TIMP1 was abolished in GANAB-depleted CALU6 cells (Supplemental Figure 11D). Together, these findings suggest that α-Glu II modulates the secretion and function of its substrates, including LH2 and TIMP1.

## Discussion

The ER plays a central role in the early secretory pathway by ensuring that nascent proteins are properly folded, glycosylated, and assembled before they are trafficked to the Golgi apparatus and beyond (11). This ER protein quality control system is critical for maintaining proteostasis and preventing the accumulation of misfolded or dysfunctional proteins. In the context of cancer, cells often experience high biosynthetic and secretory demands (25), highlighting the necessity of the machinery for cancer progression. Tumor cells rely heavily on efficient ER functions to sustain rapid proliferation and modulate the TME through secretion of cytokines, growth factors, and other glycoproteins (26). In our study, we found that N-glycan processing enzymes are frequently upregulated across multiple human cancers, including NSCLC, confirming that cancer cells exhibit an increased dependence on glycoproteins, which represents a potential vulnerability in cancer.

GANAB, which encodes the catalytic subunit of α-Glu II, is a key regulator of N-linked glycoprotein maturation (16). It catalyzes the sequential removal of glucose residues from nascent glycoproteins — a critical step required for calnexin/calreticulin-mediated folding cycles and for proper ER exit (16). In support of its broader relevance, GANAB or PRKCSH overexpression has been reported in multiple cancers — including bladder, gastric, and melanoma — and is often associated with tumor aggressiveness and poor clinical outcomes (27–29). In bladder cancer, for example, GANAB promotes cell cycle progression and invasiveness, and its depletion induces G1 cell cycle arrest (28), consistent with our observations in NSCLC. Moreover, mutations in GANAB are causative for autosomal dominant polycystic kidney and liver disease (ADPKD/ADPLD), where loss of glycosidase activity disrupts folding and trafficking of polycystin proteins (30), underscoring its essential role in ER homeostasis across disease contexts. While GANAB is broadly involved in ER protein quality control, our study reveals a previously unrecognized role for GANAB in the early secretory pathway of cancer cells. Specifically, we show that α-Glu II activity is essential for the ER-to-Golgi trafficking of select protumorigenic glycoproteins, such as LH2 and TIMP1, which are central to ECM organization (31–34). GANAB loss leads to the accumulation of these substrates within the ER and impairs their secretion, thereby compromising the tumor-promoting secretome. This highlights GANAB as a bottleneck in the secretory pathway, with selectivity toward key cancer-relevant glycoproteins.

Given its enzymatic specificity, cancer-selective function, and position at a critical checkpoint in the secretory pathway, α-Glu II represents a promising therapeutic target. Unlike global inhibitors of protein synthesis or ER function, targeting α-Glu II could selectively suppress the secretion of protumorigenic factors while minimizing systemic toxicity. Several α-Glu I and II inhibitors have demonstrated antiviral activity in vitro (35). However, their antitumor potential remains largely unexplored. We tested 2 reported selective inhibitors of α-Glu II enzymatic activity: Deoxygalactonojirimycin (DAB) and IHVR19029 (36). Unfortunately, neither compound showed significant effects on cancer cell proliferation or migration even at a high dose (50 μM) (Supplemental Figure 12), limiting their potential for in vivo evaluation. We speculate that these inhibitors may have restricted efficacy in targeting α-Glu II, possibly due to limited uptake into the ER. Future studies aimed at developing more potent α-Glu II inhibitors or approaches to reduce protein levels, such as proteolysis-targeting chimeras, may provide novel strategies to exploit secretory vulnerabilities in cancer.

A limitation of this study is that we did not characterize the N-glycan modifications of LH2 or TIMP1 by GANAB. Future studies employing mass spectrometry to analyze N-glycan alterations will be essential to determine whether impaired protein secretion is directly caused by disrupted N-glycosylation. Another intriguing yet unresolved question is how GANAB selectively or preferentially modifies oncogenic proteins. Profiling GANAB substrates using the Biotin Identification (BioID) proximity labeling method may offer valuable insights. Furthermore, we have shown that, in addition to GANAB, several other N-glycan–processing enzymes are upregulated in NSCLC and are essential for cancer cell proliferation and migration. Further studies are warranted to elucidate their roles in tumorigenesis and assess their therapeutic potential. Lastly, and importantly, we were unable to identify potent inhibitors suitable for evaluating the therapeutic efficacy and safety of targeting α-Glu II in vivo, which limits the translational implications of this study.

In summary, we demonstrate that cancer cells upregulate α-Glu II to accelerate the processing and secretion of oncogenic N-glycoproteins. These findings reveal a previously underappreciated role of N-glycan–modifying enzymes in cancer progression and provide a strong rationale for pursuing strategies to therapeutically target this pathway in cancer.

## Methods

### Sex as a biological variant.

Our study examined male mice because the syngeneic tumor model was derived using LUAD cells from male mice, and sex-mismatched tumor cells are rejected in this model.

### Mouse experiments.

For s.c. tumor models, 129/Sv mice were s.c. injected with 1 × 10^6^ murine or human lung cancer cells suspended in 100 μL phosphate-buffered saline (PBS), respectively. Orthotopic lung tumors were established by intrathoracic injection of 1 × 10^6^ murine or human lung cancer cells into 129/Sv (bred in house) or nu/nu mice (The Jackson Laboratory), respectively. Animals were monitored regularly, and necropsies were performed to measure primary tumor size and quantify metastatic lesions as previously described (37).

### Cell lines and culture.

Murine and human NSCLC cell lines (344SQ, A549, H460, H1792, H1299, H23, Calu6) were grown in a humidified atmosphere with 5% CO_2_ at 37°C in RPMI-1640 (Corning) supplemented with 10% FBS (Gibco). Human embryonic kidney (HEK) 293T cells were grown in a humidified atmosphere with 5% CO_2_ at 37°C in DMEM supplemented with 10% FBS. 344SQ is a gift from Guillermina Lozano (Department of Genetics, The University of Texas MD Anderson Cancer Center, Houston, TX). All other cells were purchased from ATCC.

### shRNAs, siRNAs, and plasmids.

The shRNAs and siRNAs targeting GANAB were purchased from Sigma. MISSION siRNA Universal Negative Control (Sigma, catalog SIC002); human siGANAB (SASI_Hs01_00120816, SASI_Hs01_00120817), murine siGANAB (SASI_Mm01_00063782, SASI_Mm01_00063783), human shGANAB (TRCN0000049578, TRCN0000049580), murine shGANAB (TRCN0000111285, TRCN0000111286). The shRNA targeting PRKCSH was purchased from sigma (TRCN0000052588). The siRNA targeting PRKCSH was purchased from MedChemExpress (catalog HY-RS11140). The pCMV3-GANAB-HA plasmid expressing human wild-type HA-tagged GANAB was purchased from Sino Biological (catalog HG18870-CY). The mouse GANAB ORF, used as a template to construct a plasmid expressing mouse wild-type GANAB, was also obtained from Sino Biological (catalog MG5A2150-U). The construct was generated using the following primers: forward: 5′-CTAGCTAGCATGGCGGCAATAGCGG-3′; reverse: 5′-CGACCGGTCGAAGATGAATACTCCAGTCG-3′. Human mutant GANAB constructs were generated by cloning into the control vector (Sino Biological, catalog CV013) using the following primers: D564N forward: 5′-TTGGAATAACATGAATGAAC-3′; D564N reverse: 5′-ACATAAAGATTAGGAGCTGA-3′, according to the manufacturer’s instructions for the Q5 Site-Directed Mutagenesis Kit (New England Biolabs, catalog E0554S). Constructs expressing Flag-tagged LH2 and GFP-tagged TIMP1 have been described previously (32).

### Lentivirus production and transduction.

Lentiviruses expressing shRNA targeting GANAB or LH2-Flag were generated in HEK293T cells using the packaging plasmids psPAX2 and pMD2.G. Target cells were infected with the viral supernatant for 48 hours, followed by selection with puromycin to establish stable knockdown or expression.

### Gene editing.

We designed 2 sgRNAs UUCAGAGCUUCUGCUUGGAA and CAUCUUCCUGAUGUCACCAU to delete the eighth exon of GANAB, which results in a frameshift and the loss of GANAB expression. We electroporated the sgRNAs (Synthego) and Cas9 protein (Integrated DNA Technologies) into the cells using the Neon electroporator (ThermoScientific). Cells were plated onto 96-well plates 48 hours after electroporation at a density lower than 1 cell per well. When the cells grew back as single-cell clones, they were expanded and screened by genomic PCR for the deletion. Genomic DNA was extracted by lysing the cells in the QuickExtract DNA Extraction solution (Biosearch Technologies), and PCR was performed using OneTaq DNA polymerase (New England Biolabs) following the standard manufacture protocol. The primers used for the genomic PCR are forward (5′-GAGGAAACACCCAGGGATGG-3′) and reverse (5′-ACCCATACAAGGCCATTGGG-3′).

### qPCR.

Total RNA was purified from cells using RNeasy Plus Kits (QIAGEN) according to the manufacturer’s protocol. The mRNA levels were quantified using a SYBRGreen-based system (Applied 20 Biosystems) after reverse transcription with qScript cDNA SuperMix (Quanta). mRNA levels were normalized based on ribosomal protein L32 (Rpl32) mRNA levels. See Supplemental Table 1 for primer sequences.

### Cell proliferation assay.

Cells were seeded in 96-well plates (2 × 10^3^ cells/well) and incubated for defined time points. Relative cell densities of 4–6 replicates per condition were measured using the WST-1 reagent (Roche).

### Cell migration and invasion assays.

Cells (2 × 10^4^ for H1299, H1975, H23 and 344SQ; 5 ×10^4^ for A549, H460 and CALU6) suspended in FBS free RPMI-1640 were seeded in the upper wells (in triplicates) of Transwell and Matrigel chambers (Falcon) and allowed them to migrate or invade, respectively, toward RPMI-1640 containing 10% FBS in the bottom wells. After 18 hours, migrating or invading cells were fixed and stained with 0.1% crystal violet (Sigma), photographed, and counted manually. Mean values were calculated from multiple fields from replicate wells.

### Flow cytometry analysis.

For apoptosis analysis, cells were collected at 80% confluency and stained using the Apoptosis Detection Kit following the manufacturer’s instructions (Thermo Fisher Scientific, catalog V13242). For cell cycle analysis, cells were collected at 80% confluency, fixed in 70% ethanol, and stained with 50 μg/mL propidium iodide (PI) (Sigma, catalog P4864) prior to flow cytometry analysis.

### RNA-seq analysis.

Triplicate samples of total RNA were obtained from parental and GANAB knockout 344SQ cells. RNA-Seq was performed by the MD Anderson Illumina Next-Generation Sequencing Core using the NovaSeq 6000 whole transcriptome sequencing protocol. The resulting RNA-seq FASTQ files were processed through FastQC, a quality control tool to evaluate the quality of sequencing reads at both the base and read levels. Samples passed QC were taken into subsequent analysis. STAR alignment was performed with default parameters to generate RNA-seq BAM files with GRCm39. Aligned reads were summarized at the gene level using STAR (Version 2.7.10b). Gene-level annotation was carried out using the GENCODE vM34 annotation, which was downloaded from the GENCODE project. The raw count data were processed by Deseq2 software to identify differentially expressed genes (DEGs) between sample groups. The final *P* value was adjusted using the Benjamini and Hochberg method. A cut-off of gene expression log_2_ fold change of ≥ 1.0 or ≤ –1.0 and an FDR *q* value ≤ 0.05 was applied to select the most significant DEGs. Raw data have been deposited to SRA database (accession no. PRJNA1428202).

### ROS measurement.

ROS production was measured according to the manufacturer’s instructions using a DCFH-DA–based detection kit purchased from Cayman (catalog 601520).

### WB analysis.

Cells were washed with PBS and then lysed to extract total proteins with cell lysis RIPA buffer (Cell Signaling Technology). Cell lysates were separated by sodium dodecyl sulfate-polyacrylamide gel electrophoresis, transferred onto Nitrocellulose Transfer Membrane (Whatman Schleicher & Schuell), and then incubated with primary antibodies (anti-GANAB (Invitrogen, catalog PA5-21431), anti-PRKCSH (Proteintech catalog 12148-1-AP), anti-tubulin (Sigma, catalog T5168), anti-PARP (Cell Signaling Technology, catalog 9542), anti-Cleaved PARP (Cell Signaling Technology, catalog 5625), anti-flag (Sigma, catalog F1804), anti-HA (Cell Signaling Technology, catalog 3724), anti-LH2 (Abnova catalog H00005352-D01P), anti-Timp1 (Proteintech, catalog 16644-1-AP), anti-Grp78 (Proteintech, catalog 11587-1-AP), and HRP-conjugated secondary antibodies (Cell Signaling Technology, catalog 7074 and 7076). Protein bands were visualized with Pierce ECL Western Blotting substrate (Thermo Fisher Scientific, catalog 34580).

### RNA polymerase II ChIP assay.

Cell lysates were formaldehyde fixed and digested using SimpleChIP Enzymatic Chromatin IP Kit (Cell Signaling Technology, catalog 9002) following the manufacturer’s instructions. Resulting chromatin was immunoprecipitated with anti–RNA polymerase II (Hologic Diagenode, catalog C15100055-100) or anti–rabbit IgG antibodies (cell signaling technology, catalog 2729). DNA was eluted and purified with the MinElute Reaction Cleanup Kit (Qiagen, catalog 28204) and subjected to qPCR. qPCR primers are listed in Supplemental Table 1.

### Immunoprecipitation assay.

Cells were lysed in cell lysis RIPA buffer. Supernatants were incubated with primary antibody and protein G agarose beads (Cell signaling Technology, catalog 9007) at 4°C overnight. The immune complex was washed with 1× RIPA buffer 4 times and boiled in 1× sodium dodecyl sulfate loading buffer at 98°C for 10 minutes. The resulting samples were subjected to WB analysis.

### miRNAs screening and Luciferase reporter assay.

miRNA mimics were purchased from Thermo Fisher Scientific, including mirVana miRNA Mimic Negative Control #1 (catalog 4464058), hsa-miR-193a-5p (MC11786), hsa-miR-133a-3p (MC10413), and hsa-miR-483-3p (MC124780). miRNA mimics including has-miR-107 (HY-R00057) and has-miR-145-5p (HY-R00282) were purchased from medChemExpress. Mimics were transfected into the indicated cell lines and incubated for 72 hours. Following transfection, cell lysates were collected for WB analysis to assess the effect of miRNA regulation on GANAB protein expression.

The GANAB 3′ UTR luciferase reporter construct (firefly luciferase) was obtained from OriGene (catalog SC211595). For the reporter assay, H1299 cells were cotransfected with miRNA mimics, the GANAB 3′ UTR reporter, and the control Renilla luciferase plasmid (pRL-TK). Luciferase activity was measured 48 hours after transfection using the Dual-Luciferase Reporter Assay System (Promega, catalog E1980), following the manufacturer’s instructions.

### LC-MS analysis.

To solubilize the CM samples, 25 μL 5% SDS and 50 mM triethylammonium bicarbonate (TEAB) (pH 7.55) were added. The solution was centrifuged at 17,000*g* for 10 minutes to remove debris. Proteins were reduced by adding 20 mM Tris(2-carboxyethyl)phosphine (TCEP) (Thermo Fisher Scientific, catalog 77720) and incubated at 65°C for 30 minutes. After cooling to room temperature, 1 μL of 0.5M iodoacetamide was added, and the solution was allowed to react in the dark for 20 minutes. Then, 2.75 μL of 12% phosphoric acid was added, followed by the addition of 165 μL binding buffer (90% methanol, 100 mM TEAB, final pH 7.1). The resulting solution was passed through an S-Trap spin column (protifi.com) using a benchtop centrifuge (30-second spin at 4,000*g*). The spin column was washed 3 times with 400 μL binding buffer. Trypsin was added to the protein mixture at a ratio of 1:25 in 50 mM TEAB (pH 8), and the solution was incubated at 37°C for 4 hours. Peptides were eluted with 80 μL 50 mM TEAB, followed by 80 μL 0.2% formic acid, and finally 80 μL 50% acetonitrile/0.2% formic acid. The combined peptide solution was dried using a speed vac and then resuspended in an autosampler vial with 2% acetonitrile, 0.1% formic acid, and 97.9% water for LC-MS analysis.

### Immunofluorescence staining.

Indicated cells were cultured on glass coverslips, fixed with formaldehyde, permeabilized with 0.5% Triton X-100 in PBS, and incubated with primary antibody (anti-Flag, anti-GFP, and anti-Calnexin; Proteintech, catalog10427-2-AP) followed by Alexa Fluor–conjugated secondary antibody (Invitrogen, catalog A-11008 and A-11011). Cells were analyzed using an A1+ platform (Nikon Instruments) confocal microscopy equipped with 60×/1.4 NA Oil, 100×/1.45 NA Oil, and 20×/0.75 NA Air objectives, 405/488/561 nm laser lines, GaAsP detectors, and Okolab stage top incubator. Images were acquired using NIS - Elements software (Nikon instruments).

### VSV-G assay.

The VSV-G transport assay was performed as described previously (38). In brief, cells were transiently transfected with EGFP-VSV-G (ts045), transferred to the restrictive temperature of 40°C for 20 hours, and then transferred to the permissive temperature of 32°C for 1 hour in the presence of 100 mg/mL cycloheximide, at which point the cells were fixed. In nonpermeabilized cells, exofacial and total VSV-G were detected by staining with an anti–VSV-G antibody and by measurement of EGFP signal intensity, respectively. VSV-G trafficking to the plasma membrane was measured based on the ratio of exofacial (surface) VSV-G fluorescence signal to the EGFP (total) signal intensity.

### RUSH assay.

The RUSH assay was performed as described previously (39, 40). In brief, cells were transiently transfected with an VSV-G reporter, which is fused to the streptavidin-binding peptide (SBP), mCherry-tag, and a second protein with a C-terminal ER retention signal (Lys-Asp-Glu-Leu; KDEL) fusing with streptavidin as a hook (Addgene, catalog 203647). Golgi-pmTurquoise2 (Addgene, catalog 36205) was transiently cotransfected to label the Golgi compartment. BioLock (IBA Life Sciences, catalog 2-0205-050) was used to quench excessive biotin in the culture medium according to manufacturer’s instructions. Twenty hours after the transfection, the release of the RUSH cargos was induced by addition of 40 μM of D-biotin (MilliporeSigma, catalog B4501). Time-lapse acquisitions were done at 37°C in a thermostat-controlled Nikon stage top incubator (Nikon Instruments).

### Microscopy.

Fixed and live-cell imaging were performed on Nikon CSU-W1 SoRa Confocal Microscope (Nikon Instruments) equipped with 60×/1.4 NA Oil, 100×/1.45 NA Oil objectives; 405/488/561/647 nm laser lines; GaAsP detectors; and Nikon stage top incubator. Images were acquired using NIS-Elements software (Nikon instruments).

### Image processing and quantitative analysis.

For fixed cell imaging, the raw images were processed, and fluorescence intensity was analyzed in Fiji/ImageJ (NIH). For live cell imaging, the raw images were processed/analyzed in Imaris 10.0.1 (Bitplane software, Oxford instruments) with Spot features and MATLAB XTensions.

### Statistics.

Results shown are representative of replicated experiments and are the mean ± SEM from at least triplicate samples or randomly chosen cells within a microscopic field. Statistical evaluations were carried out with Prism 9 (GraphPad Software, Inc.). *P* values were analyzed using Unpaired 2-tailed Student *t* tests or ANOVA for 2 or more groups, respectively, and *P* < 0.05 were considered statistically significant.

### Study approval.

All mouse studies were approved by the Animal Welfare Committee (AWC), the Institutional Animal Care and Use Committee (IACUC) of The University of Texas Health Science Center at Houston. Mice were maintained under standard husbandry conditions and euthanized at predetermined time points or upon the first signs of morbidity, in accordance with AWC guidelines.

### Data availability.

All data associated with this study are present in the manuscript.

## Author contributions

SW, XT, and GX wrote the manuscript. SW conceived, designed, executed, and interpreted molecular biology, cell biology, and in vivo experiments. XT performed and interpreted TCGA data analysis as well as supervised the entire project. JMK supervised the entire project and interpreted molecular biology, cell biology, and in vivo experiments. GX and ND conceived, designed, executed, and interpreted the cell imaging analysis. JX generated GANAB KO cells. AC, DC, KG, and YX assisted SW with cell biology assays. WKR directed and interpreted the mass spectrometry experiments.

## Conflict of interest

JMK has received consulting fees from Halozyme and Terasom Ltd.

## Funding support

This work is the result of NIH funding, in whole or in part, and is subject to the NIH Public Access Policy. Through acceptance of this federal funding, the NIH has been given a right to make the work publicly available in PubMed Central.

- NIH (7R03CA280382-02, to XT; R00 CA249048 to GX; 1R01CA251067 to JMK)
- The University of Texas Medical Branch at Galveston Mass Spectrometry Facility is supported in part by Cancer Prevention and Research Institute of Texas (CPRIT) grant RP190682 (to WKR)
- CPRIT Research Training Award (CPRIT Training Program RP210028) to AC

## Supplementary Material

Supplemental data

Unedited blot and gel images

Supplemental table 1

Supplemental table 2

Supporting data values

## Version 1 06/08/2026

Electronic publication

## Version 2 06/24/2026

"CPRIT Research Training Award (CPRIT Training Program RP210028) to AC" added to Funding support
