# Supplementary material for: Endoplasmic reticulum–resident α-glucosidase II drives non-small cell lung cancer progression via regulation of secretory glycoproteins
Source: JCI Insight. 2026 Jun 8;11(11):e203262. doi: 10.1172/jci.insight.203262 (PMC13293572; doi:10.1172/jci.insight.203262)
Supplement: Supplemental data [file jciinsight-11-203262-s350.pdf]

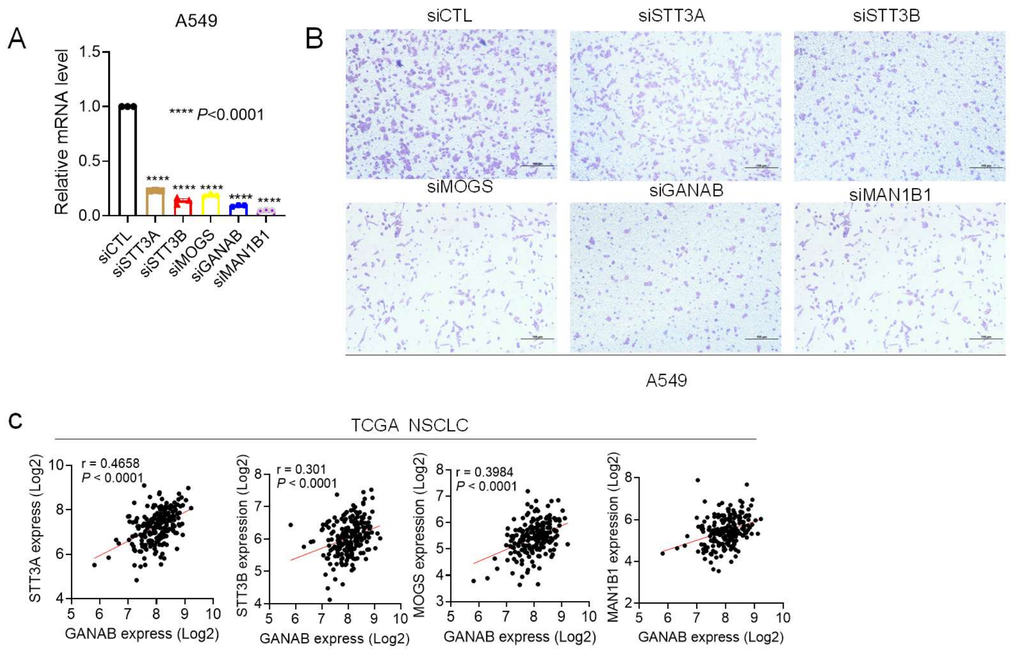

Figure S1. Genes encoding N-glycan–modifying enzymes are prognostic. A. qPCR validation of target gene knockdown in A549 cells. *P* values were determined using One-way ANOVA. B. Representative images of Boyden chamber assays. C. Pearson's correlations between GANAB and STT3A, STT3B, MOGS, or MAN1B1 mRNA levels in the TCGA NSCLC cohort. Data indicate the mean ± SEM from a single experiment incorporating biological replicate samples (*n*≥3) and are representative of at least 2 independent experiments.

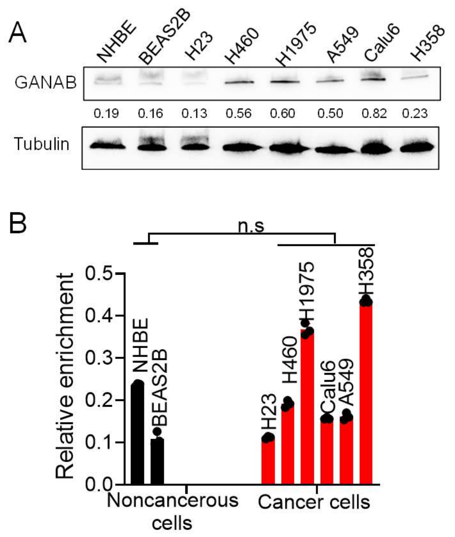

Figure S2. GANAB upregulation in cancer cells is not driven by increased transcription. A. Western blot analysis of GANAB protein levels in noncancerous lung epithelial cells (NHBE, BEAS-2B) and non-small cell lung cancer (NSCLC) cell lines (H23, H460, H1975, A549, Calu-6, H358). B. qPCR analysis of RNA polymerase II ChIP assays at the promoter region of the GANAB gene. at the GANAB promoter region. Values are normalized to GAPDH promoter signals. Data indicate the mean ± SEM from a single experiment incorporating biological replicate samples (*n*=3) and are representative of at least 2 independent experiments. *P* values were determined using Two-way ANOVA.

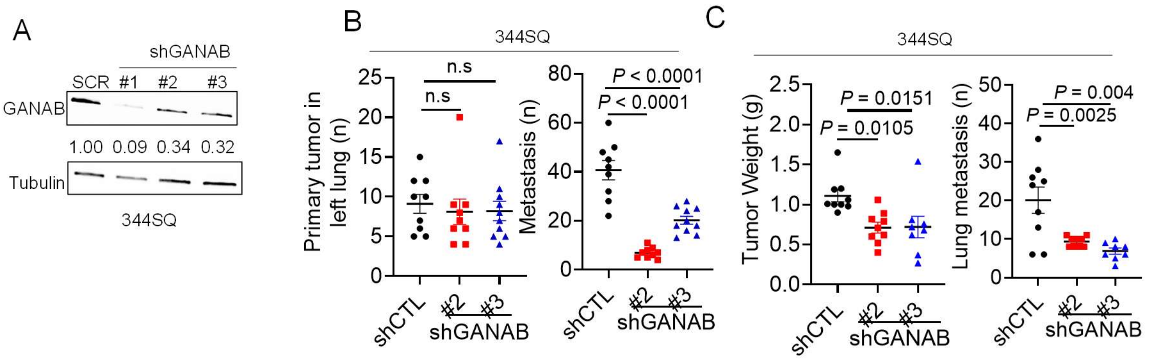

Figure S3. GANAB is essential for tumor growth and metastasis. A. WB confirmation of GANAB expression in 344SQ cells stably transfected with shCTL or shGANAB. B. Numbers of orthotopic lung tumors (left) and metastases to mediastinal nodes and contralateral lung (right) in 129/SV mice (dots represent individual mouse numbers) injected orthotopically with SCR- or shGANAB-344SQ cells. C. Flank tumor weights (left) and numbers of metastases to mediastinal nodes and contralateral lung (right) in 129/SV mice (dots represent individual mouse numbers) injected subcutaneously with SCR- or shGANAB-344SQ cells. *P* values were determined using One-way ANOVA.

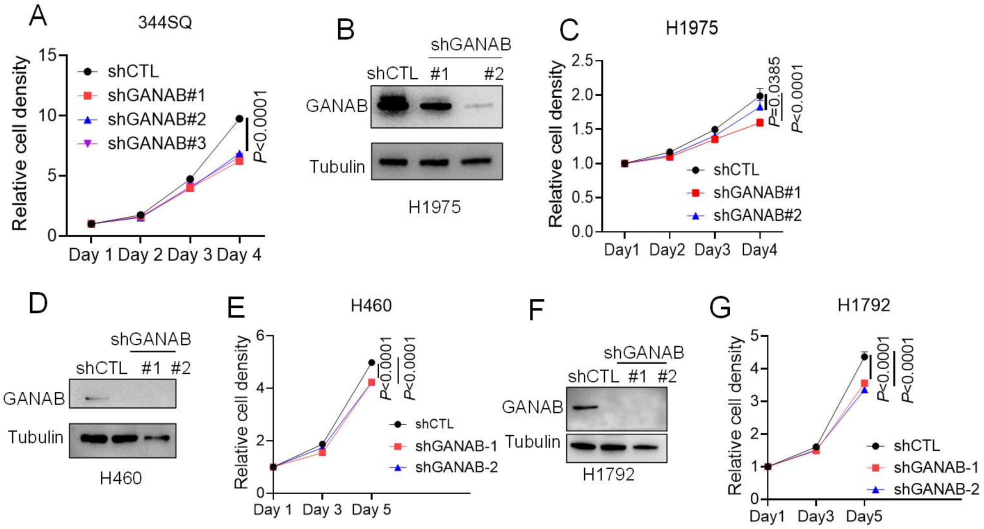

Figure S4. GANAB is essential for cancer cell proliferation. A, C, E, F, G. Relative cell densities measured by WST-1 proliferation assays in the indicated cell lines. B, D, F. WB confirmation of GANAB knockdown in the indicated cell lines stably transfected with GANAB- targeting shRNA or control shRNA. Data indicate the mean ± SEM from a single experiment incorporating biological replicate samples ($n \geq 3$) and are representative of at least 2 independent experiments. *P* values were determined using Two-way ANOVA.

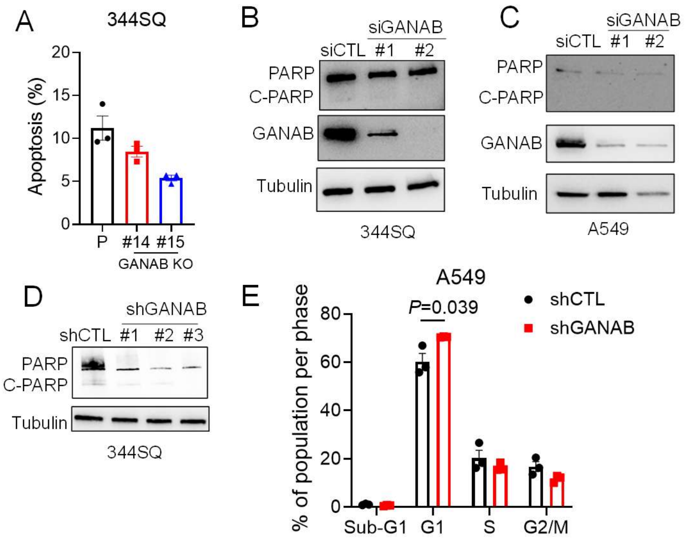

Figure S5. GANAB depletion does not induce cell apoptosis. A. Flow cytometry analysis showing the apoptosis rate in the indicated cell lines. B, C, D. WB anaylsis of PARP1, cleaved PARP1 (c-PARP) in siGANAB trasfected 344SQ (B) and A549 (C) and shGANAB-transfected 344SQ (D) cells. E. Cell cycle analysis was performed on shCTL- and shGANAB- A549 cells. Data indicate the mean ± SEM from a single experiment incorporating biological replicate samples (*n*≥3) and are representative of at least 2 independent experiments. *P* value was analyzed using two tailed Student's t-test.

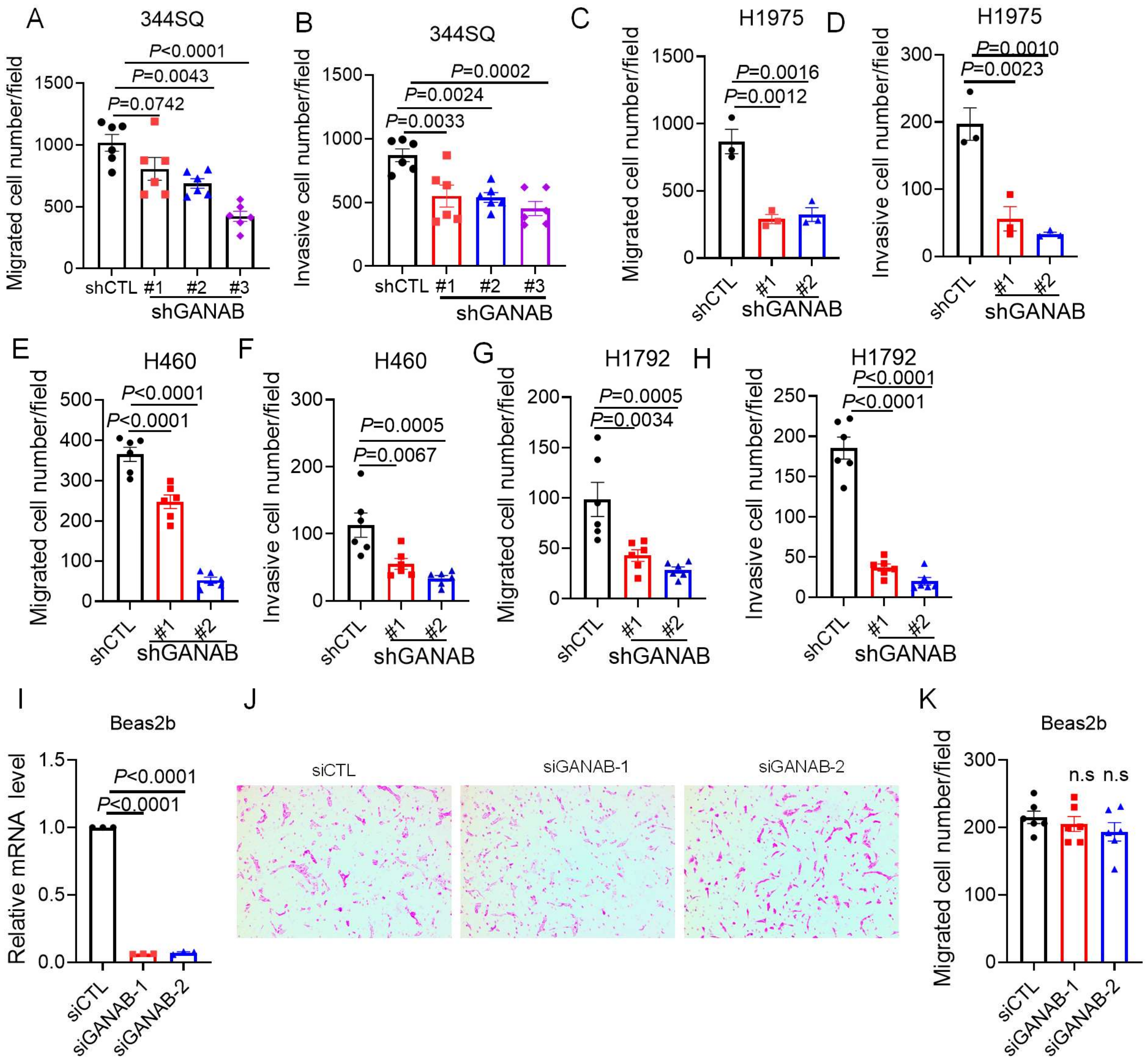

Figure S6. GANAB promotes cancer cell motility. A-H. Boyden chamber transwell assays assessing migratory (A, C, E, G) and invasive (B, D, F, H) activity in the indicated cell lines. I. qPCR validation of GANAB knockdown in Beas2b cells. J, K. Representative images (J) and corresponding quantification (K) of Boyden chamber assays assessing migratory activity in Beas2b cells. Data indicate the mean ± SEM from a single experiment incorporating biological replicate samples (*n*≥3) and are representative of at least 2 independent experiments.

P values were determined using One-way ANOVA (A-I, K).

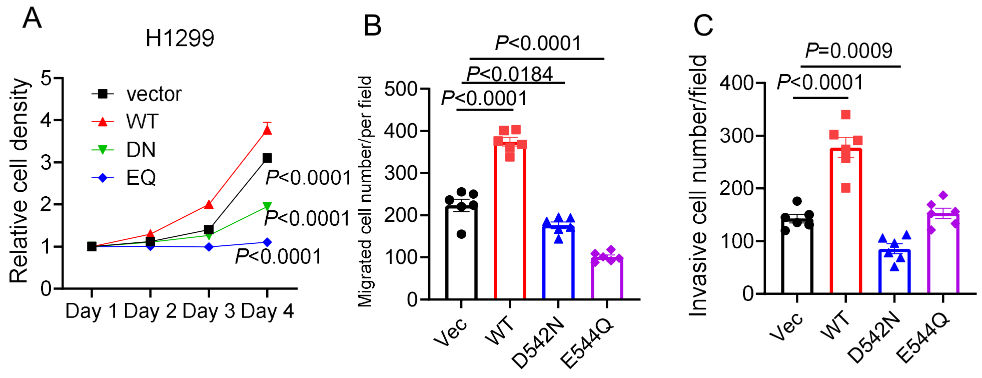

Figure S7. The Glucosidase activity of GANAB is essential to cancer cell proliferation and migration. A. Relative cell densities measured by WST-1 proliferation assays in the indicated cell lines. B, C. Boyden chamber assays assessing migratory (J) and invasive (K) activity. Data indicate the mean ± SEM from a single experiment incorporating biological replicate samples (*n*≥3) and are representative of at least 2 independent experiments.

P values were determined using Two-way ANOVA (A) or One-way ANOVA (B, C).

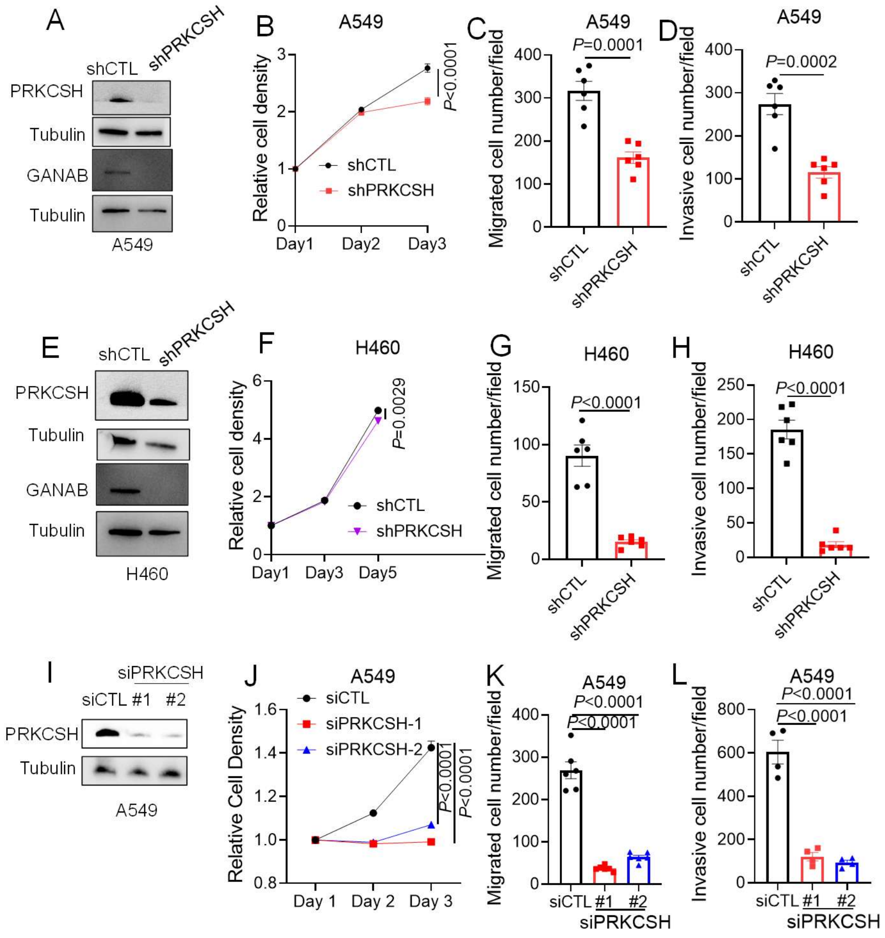

Figure S8. Depletion of PRKCSH phenocopied the effects of GANAB deficiency. A, E, I. WB analysis of PRKCSH and GANAB protein levels in the indicated cell lines. B, F, J. Relative cell densities measured by WST-1

proliferation assays in the indicated cell lines. C, G, K. Boyden chamber assays assessing migratory activity in the indicated cell lines. D, H, L. Boyden chamber assays assessing invasive activity in the indicated cell lines. Data indicate the mean ± SEM from a single experiment incorporating biological replicate samples (*n*≥3) and are representative of at least 2 independent experiments. *P* values were determined using Two-way ANOVA (B, F, J) or One-way ANOVA (C, D, G, H, K, L).

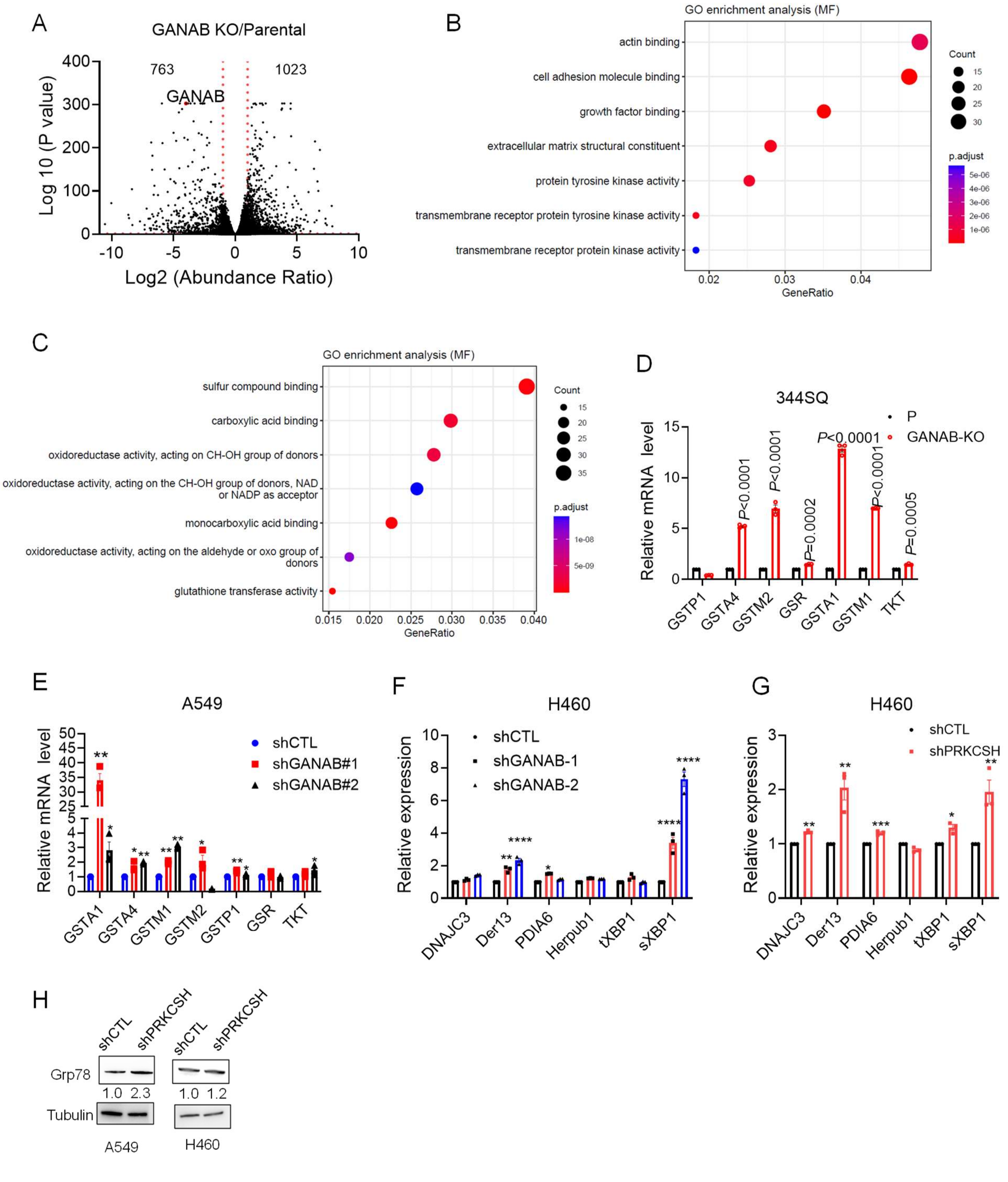

Figure S9. GANAB deficiency induces ER-stress. A. Volcano plot showing differentially expressed genes in P- and GANAB KO- 344SQ cells. Differentially expressed genes were identified based on a fold change ≥ 2 and adjusted p-value < 0.05. B.C Gene Ontology (GO) analysis of downregulated genes (B) and upregulated genes (C) in GANAB KO 344SQ cells compared to parental cells. D-G. qPCR analysis of target gene expressions in

the indicated cell lines. H. WB confirmation of target protein levels in the indicated cell lines. Data indicate the mean ± SEM from a single experiment incorporating biological replicate samples ($n \geq 3$) and are representative of at least 2 independent experiments. *P* values were determined using One-way ANOVA. **P*<0.05; ***P*<0.01;****P*<0.001; *****P*<0.0001.

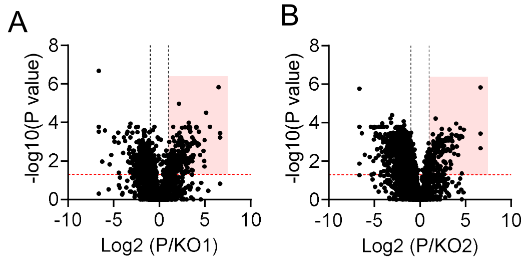

Figure S10. GANAB regulates secretion. A, B. Volcano plots of proteomic analysis by LC-MS of conditioned medium collected from parental or GANAB KO-344SQ cells. Differentially secreted proteins were identified based on a fold change ≥ 2 and adjusted p-value < 0.05.

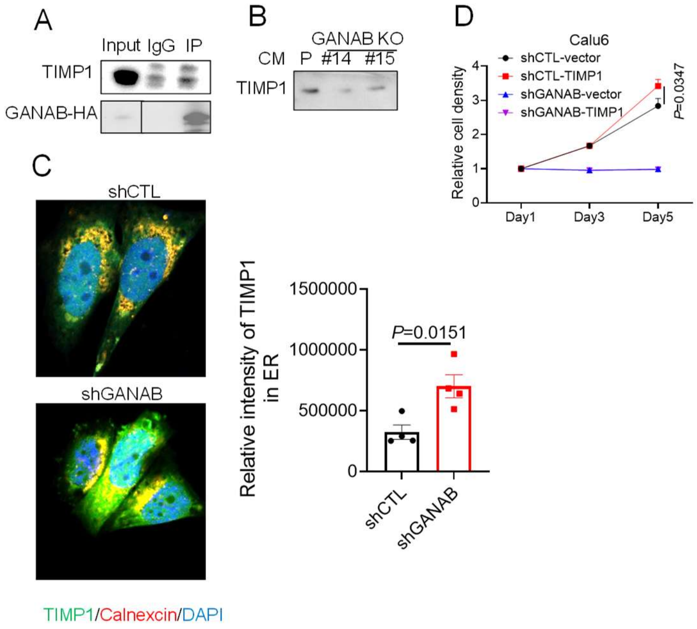

Figure S11. GANAB regulates the secretion and function of TIMP1. A. WB analysis of target proteins in Co-IP samples using GANAB-HA as bait in H1299 cells co-transfected with GANAB and TIMP1 expression constructs. B. WB analysis of TIMP1 levels in conditioned medium from parental and GANAB KO- 344SQ cells. C. Representative immunofluorescence images (left panel) showing TIMP1 (green) and calnexin (red) staining, and intensity of TIMP1 within the ER (right panel) in shCTL- and shGANAB- A549 cells. Original magnification: 40×. *P* values were determined by Student t-test. D. Relative cell densities measured by WST-1 proliferation assays under indicated condition. Data indicate the mean ± SEM from a single experiment incorporating biological replicate samples ($n \geq 3$) and are representative of at least 2 independent experiments. *P* values were determined using Two-way ANOVA.

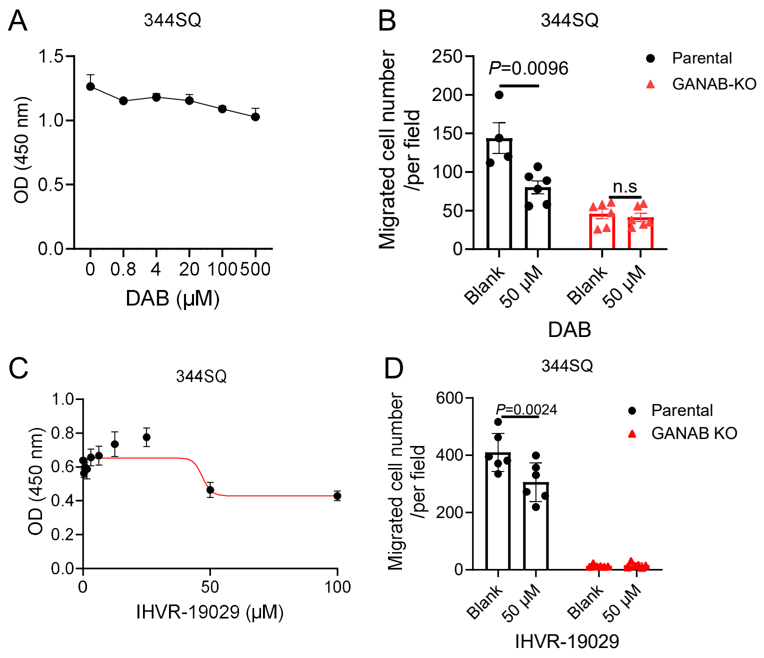

Figure S12. Commercially available glucosidase II inhibitors show suboptimal potency in cancer cells. A and C. OD values of 344SQ cells treated with DAB (A) or IHVR-19029 (C) were measured using the WST-1 reagent. B and D. Boyden chamber assays assessing migratory activity in the indicated cell lines following treatment with DAB (B) or IHVR-19029 (D). Data indicate the mean ± SEM from a single experiment incorporating biological replicate samples ($n \geq 3$) and are representative of at least 2 independent experiments. *P* values were determined using Student's t-test.
