# Supplementary material for: Endoplasmic reticulum–resident α-glucosidase II drives non-small cell lung cancer progression via regulation of secretory glycoproteins
Source: JCI Insight. 2026 Jun 8;11(11):e203262. doi: 10.1172/jci.insight.203262 (PMC13293572; doi:10.1172/jci.insight.203262)

Figure 2B

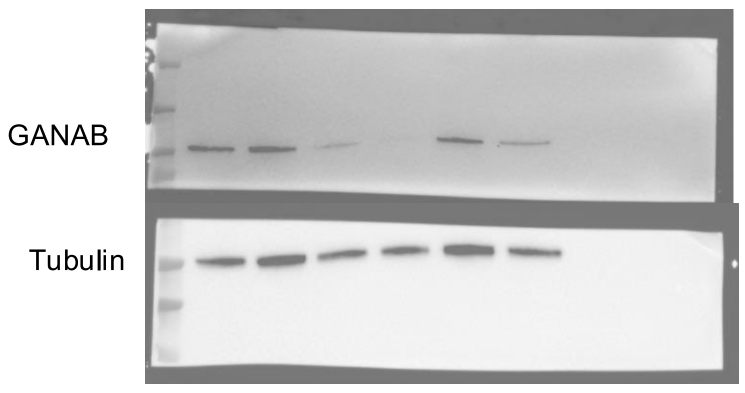

Figure 2C

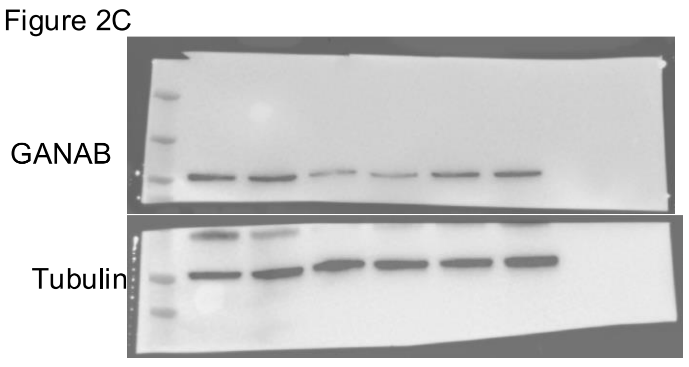

Figure 3A

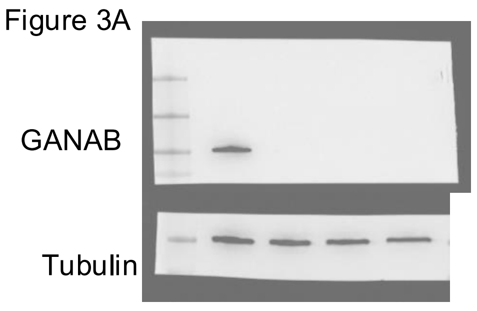

Figure 3D

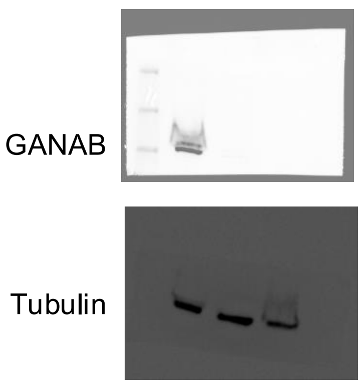

Figure 3F

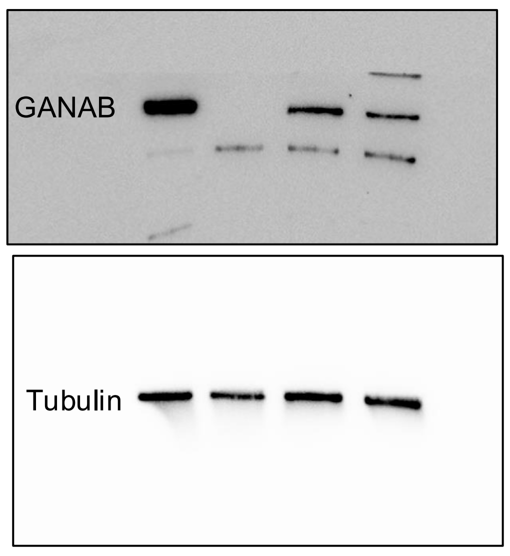

Figure 4J

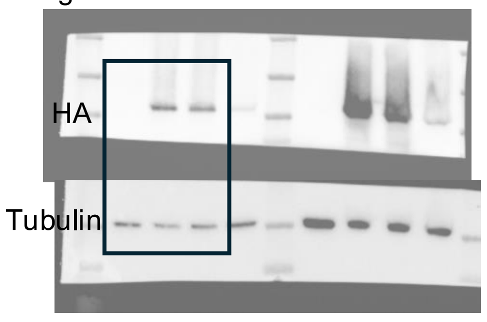

Figure 5E

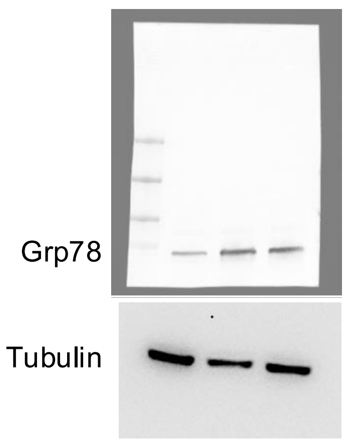

Figure 5F

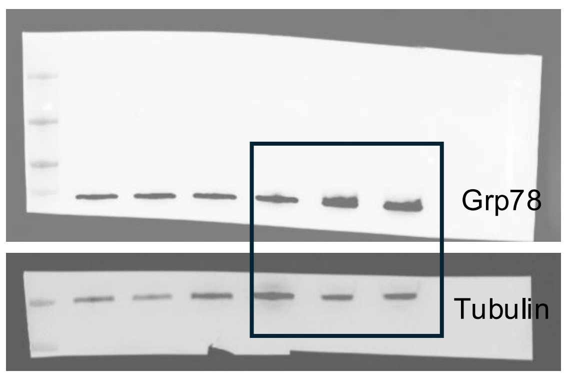

Figure 5G

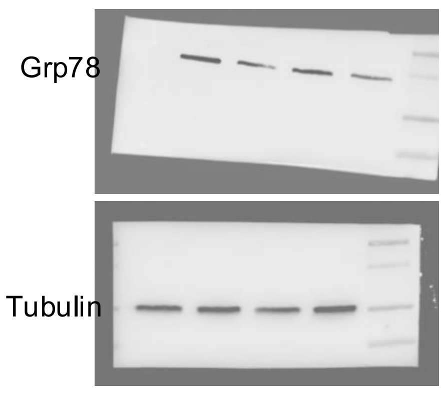

Figure 7B

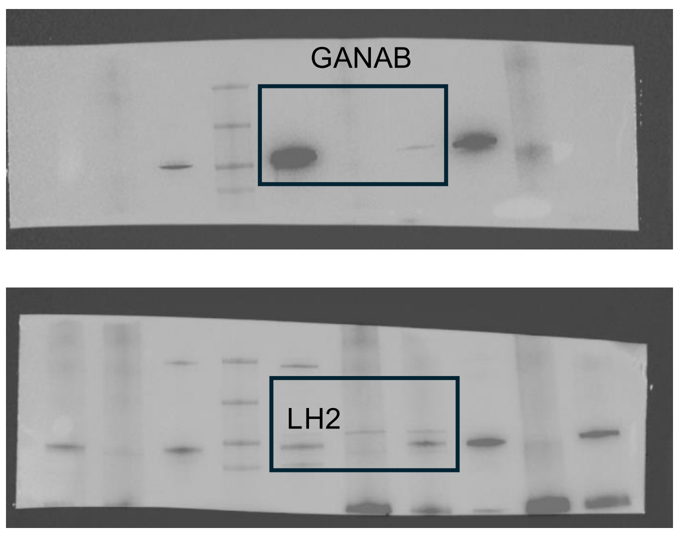

Figure 7C

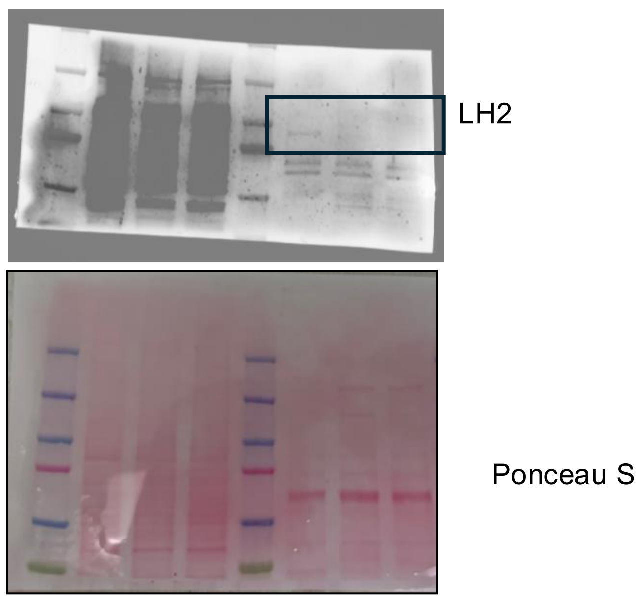

Figure 7D

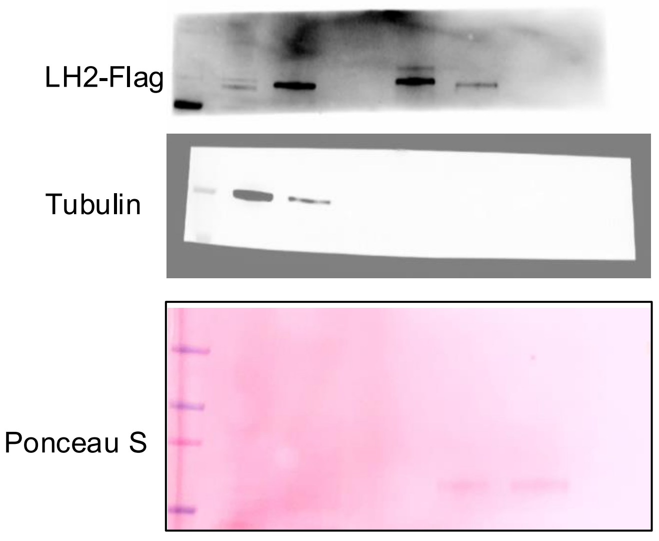

Figure 7G

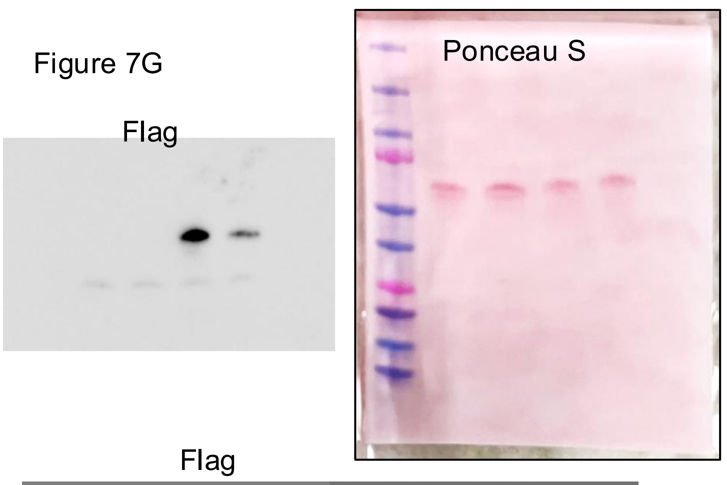

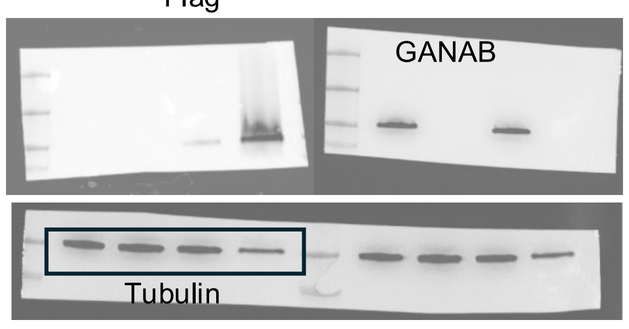

Figure S2A

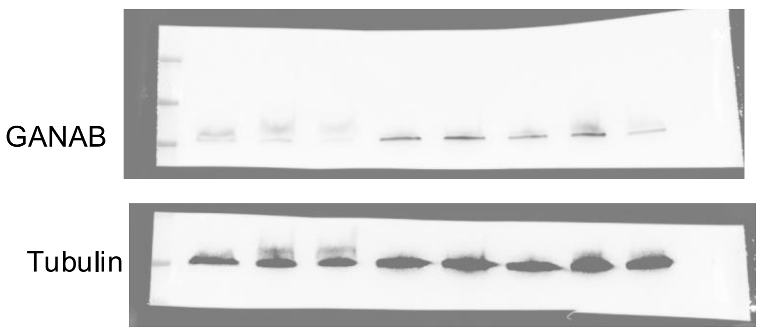

Figure S3A

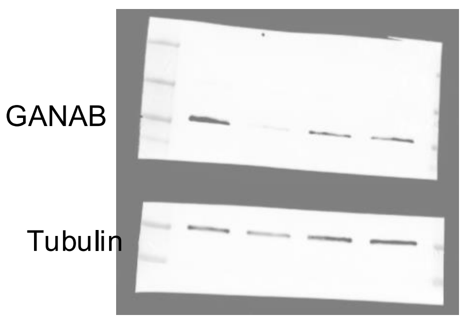

Figure S4B

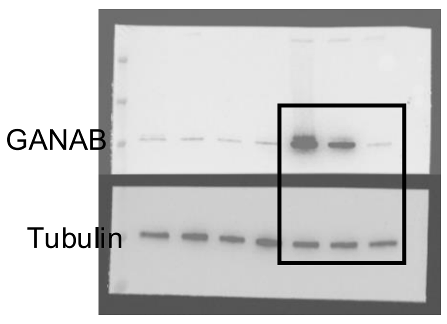

Figure S4D

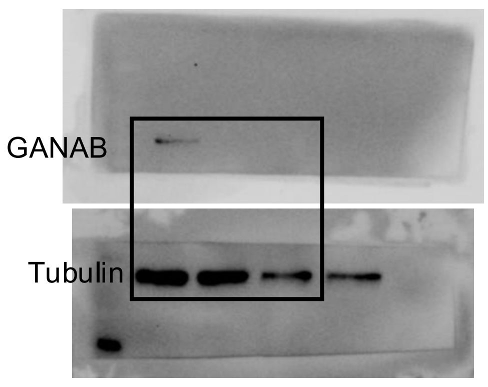

Figure S4F

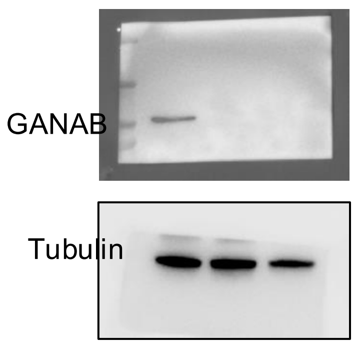

Figure S5B

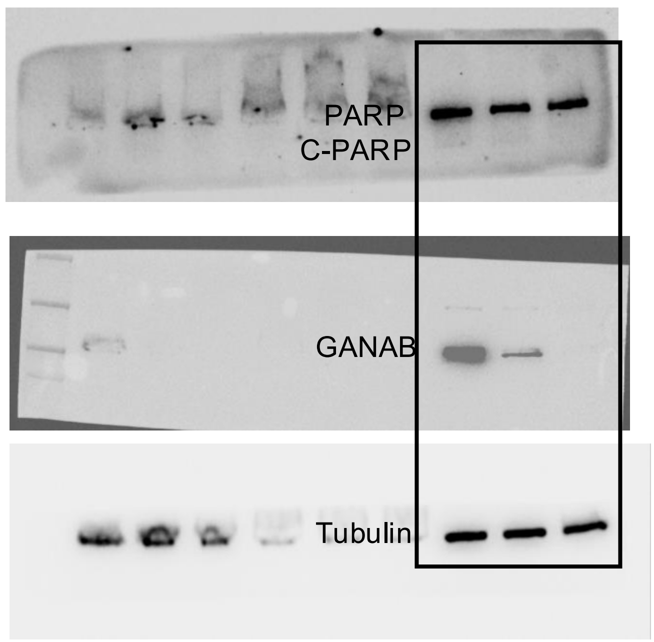

Figure S5C

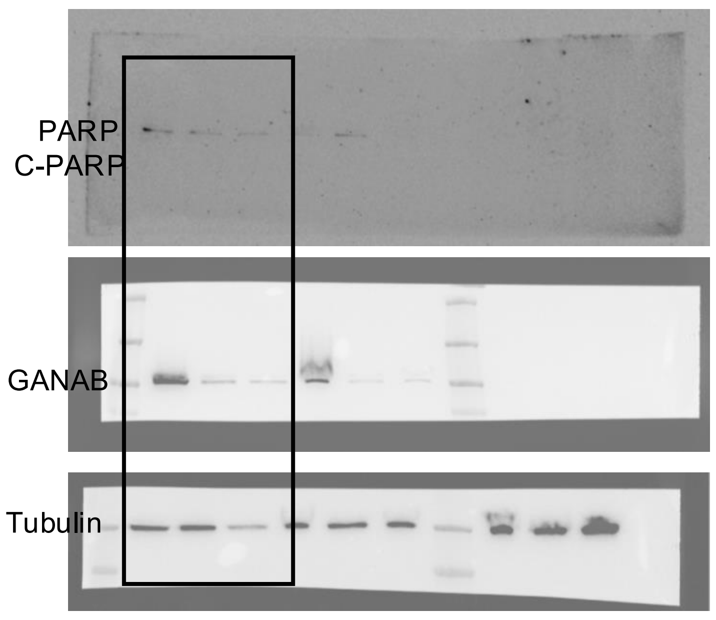

Figure S5D

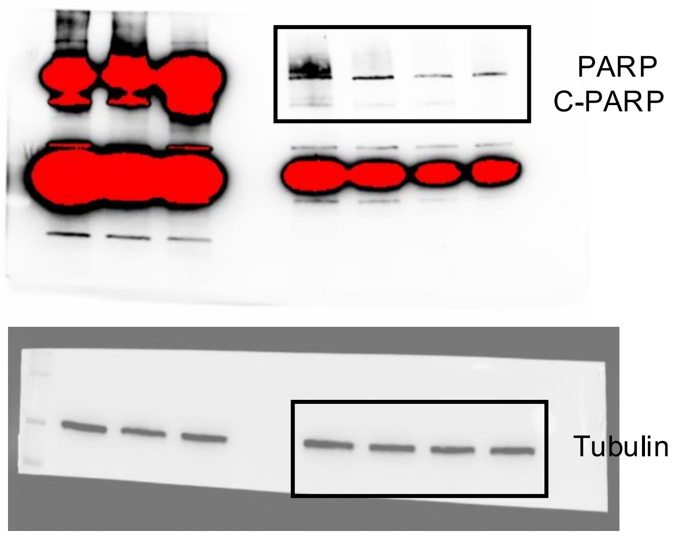

Figure S8 A and E and 9H A549 tubulin

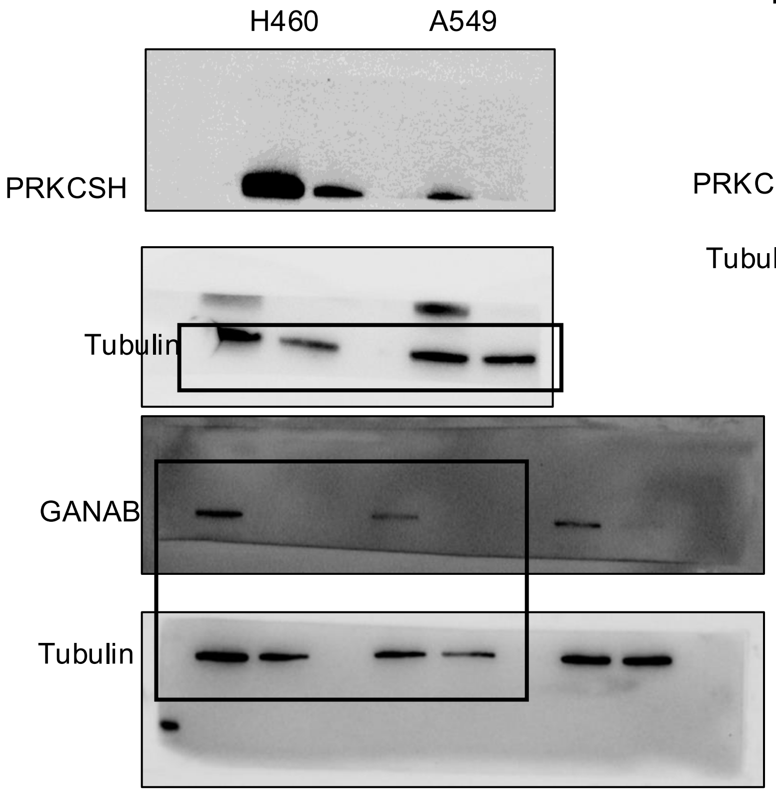

Figure S8I

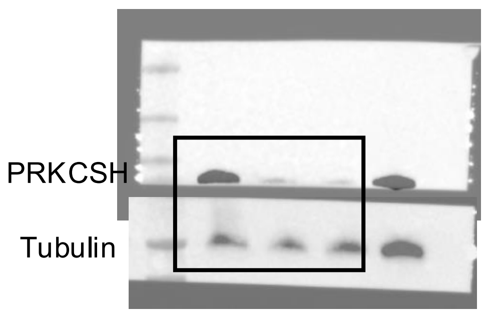

Figure S9H

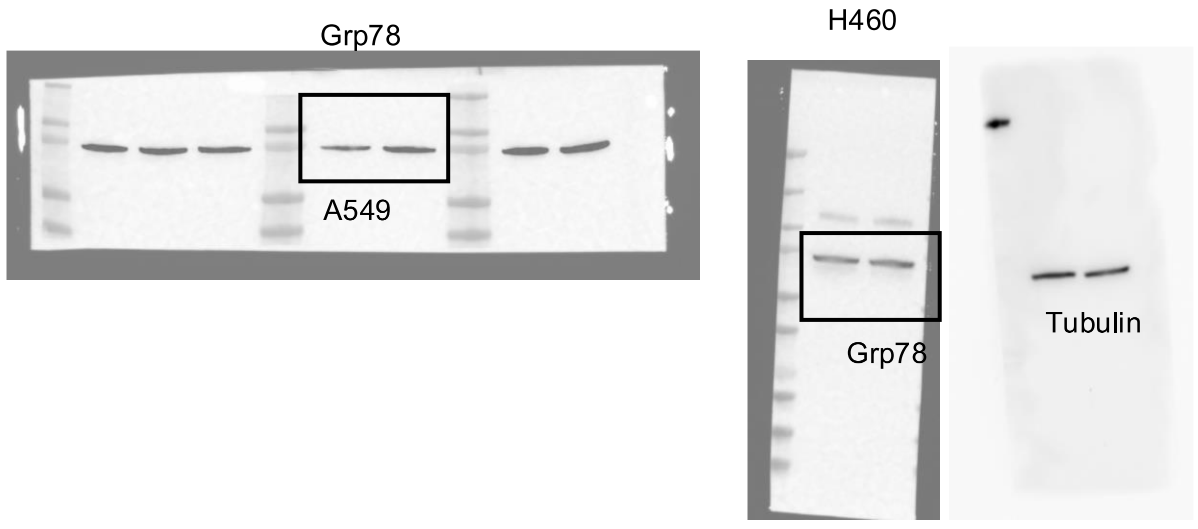

Figure S11A

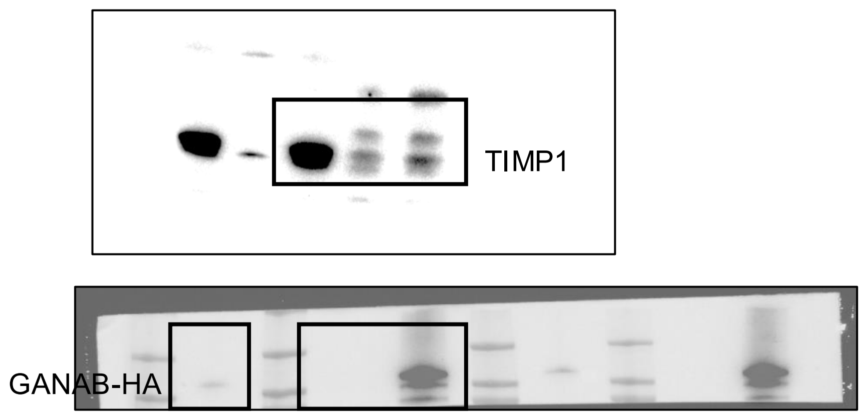

Figure S11 B

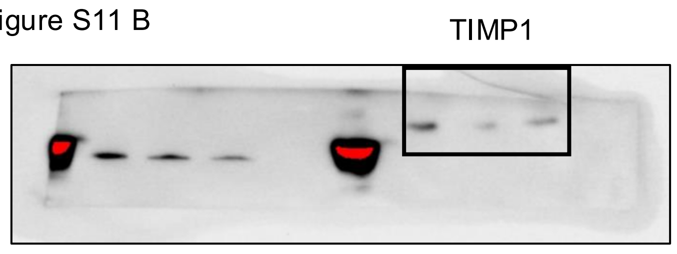

Supplement: Unedited blot and gel images [file jciinsight-11-203262-s351.pdf]
